# Functional redundancy and synthetic haploinsufficiency among Arabidopsis ATP-citrate lyase genes

**DOI:** 10.3389/fpls.2026.1961717

**Published:** 2026-10-09

**Authors:** Basil J. Nikolau, Libuse Brachova, Joel D. Schmidt, Eve Syrkin Wurtele, Xuefeng Zhao, Yan Yang, Guanghui Xiao, Ling Li

**Affiliations:** 1 Iowa State University of Science and Technology, Roy J. Carver Department of Biochemistry, Biophysics and Molecular Biology, Ames, IA, United States; 2 Department of Genetic, Development and Cellular Biology, Iowa State University, Ames, IA, United States; 3 Laurence H. Baker Center for Bioinformatics and Biological Statistics, Iowa State University, Ames, IA, United States; 4 The College of Health Humanities, Jinzhou Medical University, Jinzhou, China; 5 Department of Biological Sciences, Mississippi State University, Mississippi State, MS, United States

**Keywords:** acetyl-CoA, Arabidopsis, ATP-citrate lyase, cuticle, cytosolic, metabolism, very long chain fatty acids

## Abstract

ATP-citrate lyase (ACL) catalyzes the formation of cytosolic acetyl-CoA, which is the precursor for the generation of a variety of metabolites that have significant roles in plant growth and development. In *Arabidopsis thaliana*, ACL is encoded by three *ACLA* and two *ACLB* genes. The genetic functions of these genes were characterized by using combinations of null alleles of each subunit-gene. Individual *acla* mutants and double mutants that retained only the functional *ACLA1* or *ACLA3* gene exhibited normal growth, indicating substantial functional redundancy among the *ACLA* paralogs. These mutants showed minimal effects on cytosolic acetyl-CoA-derived metabolites, although anthocyanin content consistently increased. In contrast, *aclb1; aclb2* double mutants were not recoverable, rather embryo development was arrested at the torpedo stage, demonstrating that ACL activity is essential for embryogenesis. Moreover, *ACLB* paralogs were not functionally equivalent during vegetative growth. Plants carrying a single functional *ACLB2* allele in the absence of *ACLB1* functionality displayed a severe dwarf phenotype, whereas plants carrying a single *ACLB1* allele in the absence of *ACLB2* appeared wild type, revealing synthetic haploinsufficiency of *ACLB2*. Reduced *ACLB2* dosage also decreased cuticular wax accumulation in a gene dosage-dependent manner. Collectively, these findings demonstrate that the Arabidopsis *ACL* gene family combines paralog redundancy with subunit-specific synthetic haploinsufficiency essential for normal growth and development.

## 1 Introduction

ATP-citrate lyase (ACL) catalyzes the ATP-dependent cleavage of citrate to form oxaloacetate and acetyl-CoA. ACL was first identified in the 1950s and characterized from vertebrates sources (for review see Srere, 1975). These studies established that ACL is a component of the citrate-shuttle, which facilitates the export of acetyl-CoA equivalents from the mitochondria to the cytosol to support lipogenesis and cholesterogenesis (Feng et al., 2020; Luo et al., 2020). More recently, ACL has also been shown to localize to the nucleus, where it generates acetyl-CoA for histone acetylation (Ghisletti and Russo, 2025).

Although ACL catalytic activity had been reported in plants since the 1970’s (Mattoo and Modi, 1970; Nelson and Rinne, 1975; Fritsch and Beevers, 1979), its role in acetyl-CoA metabolism had not been extensively explored until it was molecularly characterized in the early 2000s (Fatland et al., 2000, Fatland et al., 2002; Langlade et al., 2002). The parallel characterization of the mitochondrial dicarboxylate–tricarboxylate carrier in plants (Picault et al., 2002; Dolce et al., 2014), indicates the functionality of the citrate-shuttle to support acetyl-CoA generation in the cytosol of plant cells.

Plant ACL is a hetero-octamer, composed of two dissimilar subunits, denoted as ACLA (~45 kDa) and ACLB (~65 kDa), and these are organized in an A_4_B_4_ quaternary complex (Fatland et al., 2002; Langlade et al., 2002). This contrasts with the animal ACL enzyme, which is a homo-tetramer (Feng et al., 2020). However, each animal ACL subunit is composed of two sequential domains that are homologous to the plant ACLA and ACLB subunits, respectively (Fatland et al., 2002). In the Arabidopsis genome, three genes encode the ACLA subunit: *ACLA1* (At1g10670), *ACLA2* (At1g60810) and *ACLA3* (At1g09430), and two genes encode the ACLB subunit: *ACLB1* (At3g06650) and *ACLB2* (At5g49460) (Fatland et al., 2002).

In contrast to animal cells, the plant cytosolic acetyl-CoA pool is not the precursor for the *de novo* biosynthesis of fatty acids and cholesterol, but it is a substrate for the biosynthesis of a plethora of metabolites, many of which are important for growth, development, and responses to environmental cues (Oliver et al., 2009). Specifically, these cytosolic processes use acetyl-CoA as a substrate in carboxylation, condensation or acetylation reactions, which feed into different metabolic pathways. Carboxylation of acetyl-CoA generates malonyl-CoA as an intermediate in the biosynthesis of very long chain fatty acids (i.e., 20-carbons and longer) (Zhukov and Popov, 2022), polyketides (Bisht et al., 2021), free malonate (Stumpf and Burris, 1981; Li and Copeland, 2000) and malonylated derivatives of a variety of metabolites (Martin and Saftner, 1995; Kogawa et al., 2007; Taguchi et al., 2010). Very long chain fatty acids themselves are involved in the biosynthesis of a diverse set of products, including seed oils, membrane phospholipids, sphingolipids and the cuticle (reviewed in Batsale et al., 2021). The acetyl-CoA condensation reaction generates acetoacetyl-CoA, which is the precursor of a variety of isoprenoids via mevalonate (Bach et al., 1999; Clouse, 2002; Jin et al., 2012). Additionally, acetyl-CoA can be used to acetylate other substrates, to form for example acetyl-esters (Knudsen and Tollsten, 1993) or N-acetyl-amides (e.g., Back, 2021), and some of these esters and amides occur as post-translational protein modifications (e.g., enzymes and histones) that can regulate complex processes (Verdone et al., 2006; Xia et al., 2022; Sofeo et al., 2023).

The role of ACL as a component of the citrate-shuttle that generates the cytosolic acetyl-CoA pool in plants has been evaluated with ACL mutants. Fatland et al. (2005) addressed this question by generating a series of antisense-RNA lines that down-regulated ACLA expression, resulting in a series of plant lines with a range of decreased ACL activity. Plants with even moderately reduced ACL activity developed a complex ‘bonsai’ phenotype, which includes miniaturized organs, smaller cells, aberrant plastid morphology, reduced cuticular lipid deposition, and hyper-accumulation of starch (Wang et al., 2024), anthocyanin, and stress-related mRNAs in vegetative tissue. The severity of the phenotype correlated with the degree of reduction in ACL activity, suggesting that ACL regulates the supply of cytosolic acetyl-CoA, which is required for these processes. In revelatory experiments, malonate treatment of the antisense-*ACLA* plants caused a reversion of the mutant phenotypes to a near wild type state, indicating that a decrease in cytosolic acetyl-CoA synthesis and its subsequent metabolism thru malonyl-CoA is likely responsible for the observed ‘bonsai’ phenotype (Fatland et al., 2005). In concordance, Xing et al. (2014) observed increased accumulation of cytosolic acetyl-CoA derived phytochemicals, including cuticular waxes and polyisoprenoids upon the transgenic overexpression of the ACL. These conclusions, derived from studies conducted in the model genetic system *Arabidopsis*, were further corroborated by analogous investigations in non-model species, including pepper (Yong et al., 2025) and citrus (Hu et al., 2015; Guo et al., 2020).

The current study was conducted to better understand the functional significance of each of the five *ACL* genes in plant metabolism, growth, and development. This was explored by evaluating the expression of each *ACL* gene by RT-PCR and with transgenic plants carrying *promoter:GUS* gene-fusions. In addition, we characterized plants carrying mutant alleles of each paralog, and combination of double mutants. Each of these single- and double-mutant lines were characterized both morphologically and biochemically by using variety of assays of downstream product of cytosolic acetyl-CoA metabolism. These genetic dissection of the ACL gene family demonstrate that ACL activity is essential for embryo development and that one of its subunit genes, ACLB2, revealed an unexpected dosage sensitivity within this otherwise functionally redundant gene family.

## 2 Materials and methods

### 2.1 Arabidopsis genetic seed stocks

Genetic stocks carrying T-DNA insertion or *Ds* transposon tagged alleles were identified in the SALK Institute SIGnAL T-DNA Insertion database (Alonso et al., 2003), and were obtained from the Arabidopsis Biological Resource Center (Columbus, OH), from the GABI-Kat collection (Li et al., 2007), from the INRA Versailles collection (Samson et al., 2002) or from the IMA collection (Parinov et al., 1999). Specific genetic stocks carrying mutant alleles in each ACL gene were labelled as *acla1-1* (SALK_021437), *acla2-1* (FLAG_CFN21), *acla2–2* allele (GABI_187H03), *acla3-1* (SALK_120573), *acla3-2* (SALK_067448), *aclb1-1* (GABI_895F02), *aclb1-2* (CS100148), *aclb2-1* (FLAG_EIC213) and *aclb2–2* allele (GABI_540H12). **Supplementary Figure S1** shows the molecular structure of each of these alleles as determined by sequencing the DNA that flanks both sides of the insertion element (T-DNA or the *Ds* transposable element) that caused each mutation. These flanking DNA segments were isolated by PCR amplification with primer pairs that that were specific for each *ACL* gene sequence and each insertion element (**Supplementary Table S1**).

Segregation analyses of each mutant allele were consistent with the expected Mendelian segregation ratio for a recessive non-lethal mutation. As described in the Results, the exception to this behavior was the inheritance displayed by the *acla3–1* allele, and this allele was not used for detailed molecular studies.

### 2.2 Growth of plant material

Seeds were sterilized by one of two different methods. For the biochemical analyses experiments seeds were incubated for 10 min in a solution of 0.5% bleach and 0.004% Triton X-100 in 90% ethanol, and then rinsed three times with ethanol. For the transcriptome sequencing experiments seeds were surface sterilized at room temperature by consecutively treating them for 1 min with 0.3 mL of 50% (v/v) ethanol, 10–12 minutes with 0.3 mL of 50% (v/v) bleach solution containing 1% (v/v) Tween 20 (Fisher BioReagents). Seeds were then washed with three changes of 0.3 mL sterile water. The seeds were then imbibed with 0.1% agar, and plated in 10 x 10 cm square Petri plates containing 0.8% phytoagar in half-strength Murashige and Skoog medium (Murashige and Skoog, 1962), pH 5.7, and 1% (w/v) sucrose. Stratification of the seeds was achieved by placing the plates at 4°C for 4 days, and the plates were then placed, in a near vertical position, in a growth chamber (Econaire ER1210) (Raleigh, NC) that was maintained at 22°C under constant illumination at a photon flux density of ~70-90 μmol.m^-2^.s^-1^. Seedlings were harvested from the Petri plates at 18d after transfer to 22°C, and immediately frozen in liquid N_2_ and either subjected to immediate analysis or stored at -80°C for subsequent analysis. To grow plants to maturity and collect seeds, seedlings were transplanted at 7d after imbibition to Sunshine LC mix 1 (Sun Gro Horticulture, Bellevue, WA), and grown in the same growth condition.

### 2.3 DNA isolation and characterization

Standard procedures were used for the propagation of plasmids in *Escherichia coli*, and the isolation, analysis and manipulation of nucleic acids. Double-stranded plasmid DNA templates were sequenced using an ABI 373A DNA sequencer (Applied Biosystems, Waltham, MA) at the Iowa State University Nucleic Acids Facility (www.biotech.iastate.edu/dna). Sequencing primers were the M13 universal and reverse primers, and a set of primers complementary to sequences of each of the cloned genomic sequences. Primers were designed using Primer3 (Koressaar and Remm, 2007).

### 2.4 RT-PCR analysis

RNA was isolated from 100 mg of seedling tissue using the RNeasy Kit (Qiagen, Germantown, MD). Isolated RNA (250ng) was treated with DNase I to digest contaminating genomic DNA, and used as the template for reverse transcription with the SuperScript First-Strand Kit (Invitrogen, Carlsbad, CA). PCR reactions used 10% of the reverse transcribed product as DNA template. Amplification was achieved by cycling through iterations of 94 °C for 15 s, 58 °C for 30 s, and 72 °C for 2 min, and following 36 cycles, a final extension phase for 4 min at 72 °C terminated the reactions.

### 2.5 Transgenic plants

Six transgenic *Arabidopsis* stocks were generated, each carrying one of the following *promoter:GUS* transgenes: *ACLA1:GUS, ACLA2:GUS, ACLA3:GUS, ACLB1:GUS, ACLB2:GUS*, and a promoter-less *GUS* control. Each transgene was constructed by using the 1 kb DNA fragment immediately preceding the start codon of each *ACL* gene, and this fragment was cloned into the *Hin*dIII and *Bam*HI sites of the plant transformation vector pBI101.2 (Clontech, Palo Alto, CA). The resulting plasmids were transformed into the *Agrobacterium tumefaciens* strain C58C1 (Koncz and Schell, 1986), and the resulting strains were used to transform wild type *Arabidopsis*, ecotype Col-0. Plants were transformed by dipping floral organs (Clough and Bent, 1998) into a saturated culture of *A. tumefaciens* strain harboring each transformation vector. Seeds were recovered and transgenic seeds were selected by their ability to grow in the presence of lethal doses of kanamycin. Transgenic plants were propagated to at least the T3 generation, before being subjected to histochemical analysis. The transgenes were judged to be in a homozygous state, by the inheritance of the kanamycin resistance trait in progeny from individual T2-generation plants (i.e., all recovered T3 generation individuals were resistant to kanamycin).

### 2.6 Histochemical analysis of GUS activity

GUS expression was visualized using three independent transgenic lines for each transgene construct, and plants carrying a promoter-less:*GUS* transgene were used as a negative control. Fresh plant material was incubated with a solution composed of 30 mM NaH_2_PO_4_, 50 mM Na_2_HPO_4_, 8 mM EDTA, 0.12% Triton X-100, 4 mM K_3_[FeCN_6_], 4 mM K_4_[FeCN_6_]. 3H_2_O, 20% (v/v) methanol, and 1.5 mM 5-bromo-4-chloro-3-indoyl-β-D-glucuronic acid (X-GlcA) (Jefferson et al., 1987). The GUS reaction was allowed to proceed for 1, 2, 4, 6, or 24 hours at 25°C. The reaction was quenched and the tissue was cleared of chlorophyll by incubating in 70% (v/v) ethanol for a period of 3–18 h.

### 2.7 Extraction and analysis of fatty acids

Fatty acids were extracted from either ~3.0 mg seeds or ~100 mg 18d root tissue, using a protocol based on that of (Bonaventure et al., 2003). Ten µg of nonadecanoic acid (Sigma-Aldrich, St. Louis, MO) dissolved in hexane, was applied to the sample as an internal standard and the tissue was homogenized in 10% (w/v) Ba(OH)_2_. 1, 4-Dioxane was added, and the sample was incubated at 110°C for 24 h under a nitrogen atmosphere. The sample was then acidified with 6M HCl, and fatty acids were extracted three times with aliquots of hexane. Hexane extracts were pooled, and concentrated by evaporation under a stream of nitrogen gas. An aliquot of this sample was treated with 1N HCl in methanol and incubated at 80°C for 1 h under a nitrogen atmosphere. The reaction was stopped by the addition of 1 ml of 0.9% (w/v) NaCl, and the fatty acid methyl ester products were extracted with three aliquots of hexane. Hexane extracts were pooled, and concentrated by evaporation under a stream of nitrogen gas. Samples were then silylated with a solution of 6% (v/v) of bis-trimethylsilyltrifluoroacetamide and 10% (v/v) trimethyl-chlorosilane (Shareef et al., 2006), and silylated, fatty acid methyl esters were analyzed using a GC series 6890 from Agilent (Palo Alto, CA) equipped HP-1 silica capillary column (30 m x 0.32 µm, inner diameter) using helium as the carrier gas. The GC was coupled to a 5973 Agilent mass detector. The injector was held at 250 °C, and the GC oven was initially at 140 °C and increased at 4 °C/min to 230 °C and maintained at this temperature for 3 min, and then increased at 4 °C/min to 250 °C and maintained at this temperature for 5 min. Resulting chromatograms were integrated with the use of Agilent’s HP enhanced ChemStation TM G1701 BA version B.01.00 software. Comparing acquired mass spectra with those maintained in NIST05 mass spectral library identified the analytes. The concentration of the fatty acids in the samples (moles of fatty acid/g fresh tissue mass) was calculated based on the known concentration of the internal standard.

### 2.8 Extraction and analysis of cuticular lipids

Solvent extractable cuticular lipids were isolated using a standard protocol (Perera et al., 2010) optimized for *Arabidopsis*. A known amount of hexadecane (Sigma-Aldrich, St. Louis, MO) was applied to the surface of ~100 mg of seedling tissue, which acted as an internal standard for quantification. Chloroform-soluble lipids were extracted by submerging the tissue in 1 ml of solvent for 1 min. The chloroform extract was concentrated by evaporation under a stream of nitrogen gas. The dried lipid sample was dissolved in 1 ml of acetonitrile and silylated by adjusting the solution to 6% (v/v) of bis-trimethylsilyltrifluoroacetamide and 10% (v/v) trimethyl-chlorosilane (Shareef et al., 2006). After incubation at 65 °C for 30 min, the samples were cooled, dried by evaporation under a stream of nitrogen gas, and dissolved in chloroform. Silylated lipid samples were analyzed using a GC series 6890 from Agilent (Palo Alto, CA), equipped HP-1 silica capillary column (30 m x 0.32 µm, inner diameter), using helium as the carrier gas. The injector was held at 250°C, and the GC oven temperature was initially held at 80 °C for 2 min, and then increased at a rate of 5 °C/min to 260 °C, and maintained at this temperature for 10 min, and then increased at 5 °C/min to 320 °C, and maintained at this temperature for 10 min. The GC effluent was coupled to a 5973 Agilent mass detector. Resulting chromatograms were integrated using the Agilent’s HP enhanced ChemStation TM G1701 BA version B.01.00 software. Comparing acquired mass spectra with those maintained in NIST05 mass spectral library identified the analytes. The concentration of the lipids in the samples (moles/g fresh tissue mass) was calculated based on the known concentration of the hexadecane internal standard.

### 2.9 Quantification of anthocyanins

Anthocyanin content was assessed by previously published procedures (Rabino and Mancinelli, 1986; Bariola et al., 1999). Specifically, the aerial tissues from seedlings were harvested and incubated for 2 h at room temperature in 2.5 ml of 1% (v/v) HCl in methanol. Interfering pigments (i.e., chlorophylls) were removed by adding 2 ml chloroform and the samples were vortexed for 1 min. Deionized water (5 ml) was added, and the samples were again vortexed for 1 min, and centrifuged (5 min at 1500 g) to separate the phases. The absorbance at 530 nm and 657 nm of the upper, methanol/water phase was determined. Anthocyanin concentration was calculated from the difference in absorbance (A_530_ - A_657_).

### 2.10 Isolation of RNA and RNA-seq analysis

RNA-Seq analysis was performed as previously described (Li et al., 2015). RNA was isolated from 0.5 g of *Arabidopsis* seedlings using TRIzol (Invitrogen). The isolated RNA was further purified using the RNeasy PlantMini Kit (Qiagen) followed by DNase I (Invitrogen) treatment to eliminate contaminating DNA. RNA samples isolated from three biological replicates of each genotype were each used for the construction of 200-bp short insert library and these were sequenced using the Illumina HiSeq 2000 system and V3 Reagent (www.illumina.com) (BGI Americas Corporation, Cambridge, MA). Data files have been deposited in the NCBI Sequence Read Archive under accession number PRJNA1495842.

Data collected from 18 DAI and 24 DAI seedlings were analyzed independently, each as a randomized complete block design with three blocks corresponding to seedlings grown on three different shelves of a growth chamber. For both time points, one plate of each of the wild-type genotype, both homozygous mutants (i.e., genotypes *aclb1/aclb1*; *ACLB2/ACLB2* and *ACLB1/ACLB1; aclb2/aclb2*), and both heterozygous mutants (i.e., genotypes *aclb1/aclb1*; *aclb2/ACLB2* and *aclb1/ACLB1; aclb2/aclb2*) were sequenced from each of the three growth chamber shelves. Sequencing quality metrics, including raw and clean sequence yields, Q20 and GC contents, uniquely mapped read counts, and overall alignment rates to the *A. thaliana* TAIR10 reference genome, are summarized for all 30 RNA-seq libraries in **Supplementary Table S2**. Processed data that quantified the expression of each *Arabidopsis* gene as uniquely mapped raw counts per gene are presented in **Supplementary Tables S3**, **S4** presents the expression of each *Arabidopsis* gene as fragments per kilobase of transcript per million mapped reads (FPKM) per gene. These transcriptomics data were used to visualize the differences among genotypes by Principal Component Analysis (PCA), performed with the R package, FactoMineR and plotted using the R package, ggplot2.

Differentially expressed genes, between each mutant and the wild type, were identified using the R package QuasiSeq (http://cran.r-project.org/web/packages/QuasiSeq). In these comparisons, the negative binomial QLShrink method implemented in the QuasiSeq package was used to compute a p-value for each gene for each genotype comparison (Lund et al., 2012). Using the p-values for each genotype comparison, the approach of Nettleton et al. (2006) was used to estimate the number of genes with true null hypotheses among all genes tested, and this estimate was used to convert the p-values to q-values (Storey, 2002). To obtain approximate control of the false discovery rate at 5%, genes with q-values no larger than 0.05 were declared to be differentially expressed.

## 3 Results

### 3.1 Transgenic *promoter:GUS* analysis of the expression of individual *ACL* genes

Promoter segments (~1-kb DNA fragment upstream of the transcribed regions) from each of the three *ACLA* and two *ACLB* genes were fused to the *GUS* reporter gene, and the resulting constructs were transformed into Arabidopsis. The ability of each promoter to drive gene expression was monitored by GUS histochemical assays with 18-day old seedlings (**Figure 1**; **Supplementary Figure S2**). As negative controls for these experiments, both promoter-less GUS transgenics (**Figure 1F**) and non-transgenic control plants (**Supplementary Figure S2I**) showed no detectable GUS expression, even with extended period of incubation with X-GlcA.

**Figure 1:**
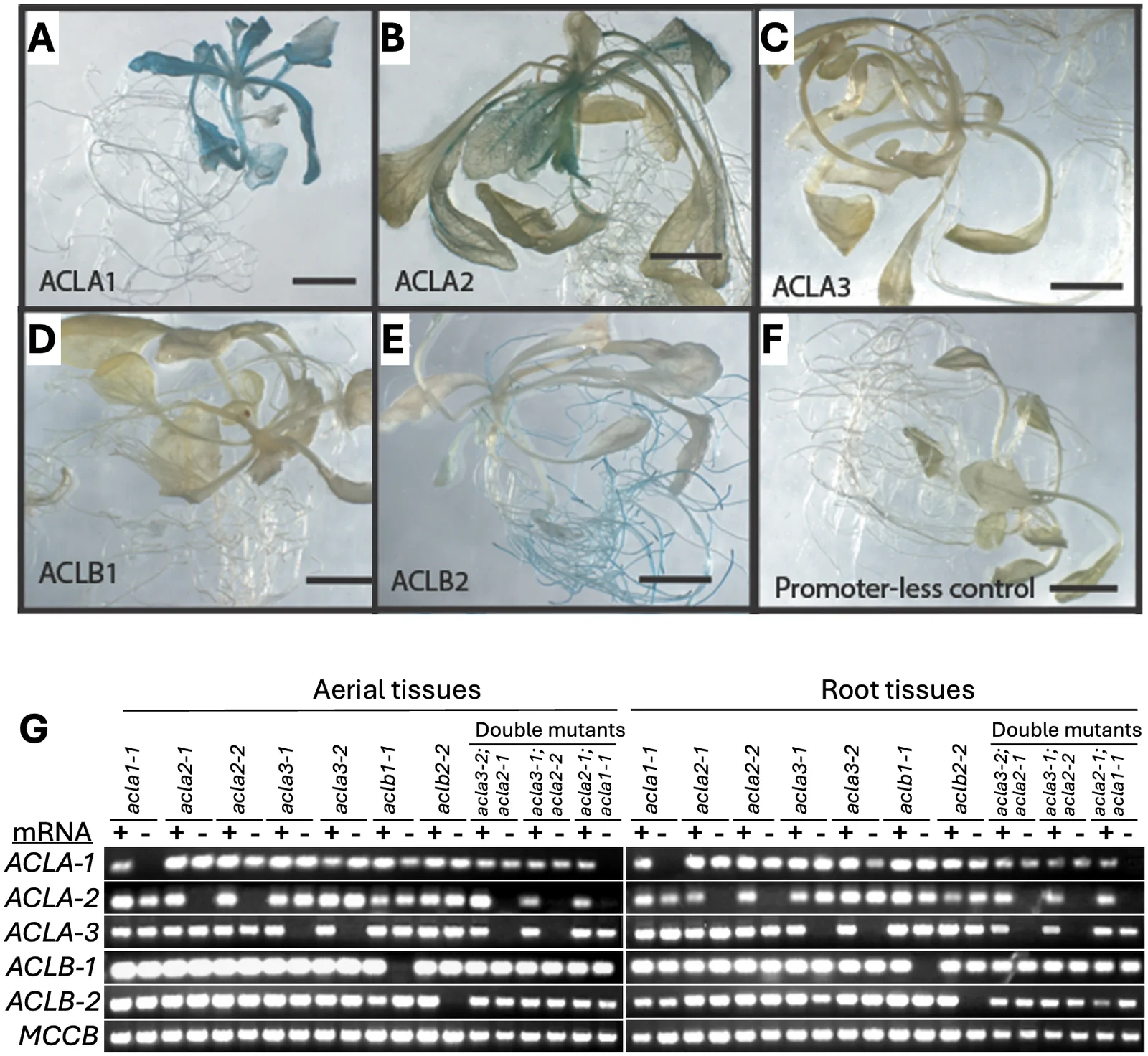
Expression of *ACL* genes. A-F. Histochemical localization of *ACL-*promoter-mediated GUS expression in 18-d old *Arabidopsis* seedlings. GUS activity was visualized by infiltrating seedlings with 5-bromo-4-chloro-3-indolyl-β-D-glucoronic acid, and incubating them for either 4 h **(A)** or 24 h **(B–F)**. GUS expression was undetectable in seedlings carrying a promoter-less *GUS* control **(F)**. Scale bars = 5 mm. **(G)** RT-PCR analysis, with gene-specific primers detect the *ACLA1*, *ACLA2*, *ACLA3*, *ACLB1*, and *ACLB2* mRNAs in aerial and root tissues of 18 d-old wild type (+) and homozygous mutant (-) sibling plants; analysis of the *MCCB* mRNA (McKean et al., 2000) served as a loading control. See **Supplementary Table S1** for primer sequences.

Significant differences were observed in GUS expression patterns, transcriptionally controlled by each of the three *ACLA* gene promoters. The expression of *ACLA1:GUS* is clearly the highest among the three *ACLA* genes (cf., **Figures 1A-C**), with highest expression being detected in the cotyledons, rosette leaves and leaf petioles (**Supplementary Figure S2A–C**). *ACLA2:GUS* expression is more discreet (**Figure 1B**), with significant expression being concentrated in the root tips (**Supplementary Figure S2D**), and vasculature and trichomes of rosette leaves (**Supplementary Figure S2E**). Low levels of *ACLA3:GUS* expression was only detectable upon longer incubation with X-GlcA, and this appears to be concentrated in the roots (**Figure 1C**; **Supplementary Figure S2F**).

Comparing the tissue expression patterns between the two *ACLB* genes indicates that *ACLB2:GUS* is expressed at higher levels than *ACLB1:GUS* (c.f., **Figures 1D, E**). Upon longer incubation with X-GlcA, *ACLB1:GUS* expression was particularly detectable in trichomes (**Supplementary Figure S2G**), whereas *ACLB2:GUS* occurs throughout the roots (**Supplementary Figure 2H**).

These distinct expression patterns displayed by each *ACL* paralog were further explored by examining the publicly available *Arabidopsis* expression atlases, i.e., the Arabidopsis eFP browser (Sullivan et al., 2019) and the Arabidopsis RNA-seq Database (Zhang et al., 2020). Although directly comparing the expression of the *ACL* genes in these datasets is difficult because the data were taken from a variety of different developmental stages, yet both the Arabidopsis RNA-seq data (**Supplementary Figure S3**) and the eFP browser data (**Supplementary Figures S4**–**S7**) provide partial confirmation of the *promoter*:GUS expression data. For example, in all three datasets, the *ACLA1* gene shows the highest expression level, and the *ACLB2* gene is expressed at higher levels than *ACLB1*. Additionally, as maybe expected for genes that encode an enzyme at the core of cytosolic metabolism, all *ACL* genes are expressed at detectable levels in all organs, with the exception of maturing siliques.

### 3.2 Molecular characterization of *acl* mutants

Insertional mutant alleles of the *ACL* genes were identified in the SALK Institute SIGnAL database (Alonso et al., 2003). The initial genetic stocks obtained from the ABRC stock center were in the heterozygous state, and these were self-propagated for three generations, while tracking the inheritance of each allele by PCR-genotyping (**Supplementary Figure S1**). For each mutant allele, several individual sibling families were generated from a single heterozygous plant, and these were PCR-genotyped to identify homozygous mutant lines for each *ACL* locus.

The expression of each *ACL* gene was assayed by semiquantitative RT-PCR using RNA preparations isolated from siblings that were homozygous mutant or wild type. All five *ACL* mRNAs were detectable in the wild type plants, but were undetectable in the sibling plants that were in the homozygous mutant state (**Figure 1G**). Therefore, we considered all isolated *acl* mutants as being null alleles. Moreover, the mRNA of only the mutated *ACL* gene was missing in each genetic stock, and there are no apparent compensatory alterations in the expression of the mRNAs of the other wild type *ACL* genes that were present in each of these mutant stocks.

### 3.3 Segregation of *acla* mutant alleles and functional redundancy among the *ACLA* genes

Inheritance of the five *acla* mutant alleles was determined by genotyping progeny siblings from heterozygous *acla* mutant stocks. Data presented in **Table 1** establish that with four of the mutant alleles (i.e., *acla1-1, acla2-1, acla2-2*, and *acla3-2*), the genotypes of the progeny were recovered at rates that are consistent with the expected Mendelian segregation ratio for a recessive non-lethal mutation. Namely, these alleles were recovered in a ratio that is statistically indistinguishable from the expected 1:2:1 ratio of homozygous wild type-heterozygous-homozygous mutant genotypes.

**Table 1:** Segregation analysis of *acla* mutant alleles.

Heterozygous parental genotype^1^	Genotype of recovered progeny^2^			Statistical test^3^		
	+/+	+/−	− −	χ^2^	α = 0.05	d.f.^4^
*acla1-1*	11	24	11	0.087	5.991	2
*acla2-1*	9	24	11	0.545	5.991	2
*acla2-2*	9	13	7	0.586	5.991	2
*acla3-1*	16	31	3	9.640*	5.991	2
*acla3-2*	16	29	9	2.111	5.991	2

^1^ The mutant allele carried in a heterozygous state by the parent.

^2^ Number of progeny recovered from the self of a heterozygous parent. Genotype classifications are homozygous wild-type (+/+); heterozygous (+/−); homozygous mutant (−/−).

^3^ Statistical χ^2^ test of the null hypothesis that the mutant allele segregates at the Mendelian ratio (1:2:1) for a recessive non-lethal mutation.

^4^ d.f., degrees of freedom.

The exception to this behavior was the inheritance displayed by the *acla3–1* allele. In this family, homozygous *acla3–1* mutant progeny were significantly under-represented, which indicated a possible issue with the transmission of the *acla3–1* allele. This potential complexity was explored by conducting an allelism-test between *acla3–1* and *acla3–2* mutants. PCR-genotyping analysis of the recovered progeny indicated that the two alleles segregated in a normal Mendelian ratio, suggesting no bias against the transmission of either mutant allele (**Supplementary Table S5**). We concluded therefore that the defective transmission phenotype displayed by the *acla3–1* stock is associated with a mutation at another genetic locus that is carried by this stock. Therefore, all subsequent analyses of the *ACLA3* gene used only the *acla3–2* mutant stock.

Plants homozygous for the *acla* mutant alleles were morphologically indistinguishable from the wild type siblings, indicating that the three *ACLA* genes are each functionally redundant, with any two *ACLA* genes being capable of compensating for a mutation in the third *ACLA* gene. This functional redundancy was further explored by the generation of double mutant stocks. Specifically, we recovered homozygous *acla2; acla3* double mutants (recovered from both the *acla3-1* ♂ × *acla2-2* ♀, and *acla3-2* ♂ × *acla2-1* ♀ crosses) and *acla1; acla2* double mutants (recovered from the *acla2-1* ♂ × *acla1-1* ♀ cross). Each of these recovered homozygous double mutant stocks expressed only a single *ACLA* gene. Specifically, only the *ACLA1* gene was expressed in the *acla2; acla3* double mutant, and only the *ACLA3* gene was expressed in the *acla1; acla2* double mutant. All of these double mutant plants were fully viable, and were free of any observable morphological growth abnormalities. Therefore, these findings amplify the functional redundancy between these two *ACLA* genes, indicating that, at least in the controlled laboratory conditions used in this study, either *ACLA1* or *ACLA3* can solely support physiological functions required for growth, development and viability.

The same could not be assessed for the *ACLA2* gene, because the *acla1; acla3* double mutants were not recoverable due to the technical limitation associated with the fact that the *ACLA1* and *ACLA3* genes are situated within 0.5 Mb of each other, on Chromosome I. Namely, recovery of a genetic recombination event between these two loci requires evaluating a very large number of progeny from a *acla3* × *acla1* cross. Therefore, we are not able to assess whether the *ACLA2* gene can solely support physiological functions required for growth, development and viability.

### 3.4 Segregation of *aclb* mutant alleles and functional redundancy between the *ACLB* genes

An analogous approach was used to investigate the inheritance and phenotypes associated with the *aclb* mutant alleles. In this case, because there are only two *ACLB* loci, we took a strategy of selfing plants that were heterozygous for both the *aclb1–1* and *aclb2–1* alleles (i.e., *ACLB1-1/aclb1-1; ACLB2-1/aclb2–1* genotype) and PCR genotyped 280 recovered progeny. Nine possible genotypes are recoverable among this pool of progeny plants, and **Table 2** compares the number of plants recovered in each genotype-category to the theoretical expectation based on Mendelian segregation of two genetically unlinked, recessive non-lethal mutations. Based on the statistical χ^2^ test, this null hypothesis is rejected, and the anomaly that explains this rejection is the absence of any double mutant plants (i.e., *aclb1-1/aclb1-1; aclb2-1/aclb2-2*). However, this χ^2^ analysis of the other genotypes indicate that they were recovered at the expected ratio (χ^2^ = 4.08, with 7 degrees of freedom, a = 0.05, and p-value=0.77, the critical χ^2^ = 14.4), indicating that the transmission of either *aclb1* or *aclb2* mutant alleles occurs normally through gametogenesis, and individual homozygous *aclb1* or *aclb2* mutant plants are viable. But the inability to recover *aclb1-1; aclb2–1* double mutant indicates that the lack any *ACLB* expression is lethal, thereby establishing that ACL catalytic activity is essential for viability.

**Table 2:** Segregation analysis of *aclb* double mutants^1^.

	Number of plants		
Genotype	Recovered^2^	Expected	
*ACLB1-1/ACLB1-1; ACLB2-1/ACLB2-1*	15	17.5	
*ACLB1-1/ACLB1-1; ACLB2-1/aclb2-1*	39	35	
*ACLB1-1/ACLB1-1; aclb2-1/aclb2-2*	22	17.5	
*ACLB1-1/aclb1-1; ACLB2-1/ACLB2-1*	42	35	
*ACLB1-1/aclb1-1; ACLB2-1/aclb2-1*	71	70	
*ACLB1-1/aclb1-1; aclb2-1/aclb2-2*	30	35	
*aclb1-1/aclb1-1; ACLB2-1/ACLB2-2*	21	17.5	
*aclb1-1/aclb1-1; ACLB2-1/aclb2-1*	40	35	
*aclb1-1/aclb1-1; aclb2-1/aclb2-2*	0	17.5	
Total of all genotypes	280		

^1^ Genotype data from the selfed progeny from parents of the double heterozygote, *ACLB1-1/aclb1-1; ACLB2-1/aclb2-1.*

^2^ χ^2^ statistical analysis rejects the null hypothesis that the mutant alleles each segregate independently with Mendelian ratio for two recessive non-lethal mutations, each at unlinked genetic loci (χ^2^ = 23.0, with threshold of 15.5 for α=0.05 and 8-degrees of freedom (p-value=0.003).

The lethality associated with the *aclb1; aclb2* double mutant was further explored by examining the seeds developing within siliques of selfed double heterozygous plants (i.e., *ACLB2/aclb2-1; ACLB1/aclb1–1* genotype). Siliques on these plants were developmentally staged by their positions on the bolt. **Figure 2** shows a schematic of the positions of the siliques from which data were collected, with position #1 marked with a red asterisk being the earliest developmental stage. A distinct phenotypic difference in the appearance of some of the ovules within each silique became apparent in siliques at position #4, which were estimated to be at 5 days after flowering (**Figures 2A, B**). These distinguishing phenotypic features of the ovules became more significant within siliques at position #5 (**Figures 2C, D**) and position #6 (**Figures 2E, F**), and a subset of ovules in siliques at position #10 were shriveled and brown (**Figures 2G, H**). In contrast, ovules in siliques dissected from wild type siblings showed no such abnormalities (**Figure 2I**). We collected such observations from 22 siliques from multiple plants, and this phenotype occurred in 45 out of 554 propagules that were examined. This frequency corresponds to the 1:16 ratio expected for *aclb1; aclb2* double homozygous mutant progeny from a double heterozygous parent (χ^2^ = 3.316, α = 0.05, 1 degrees of freedom), and we therefore surmise that these are *aclb1; aclb2* double mutant progeny ovules.

**Figure 2:**
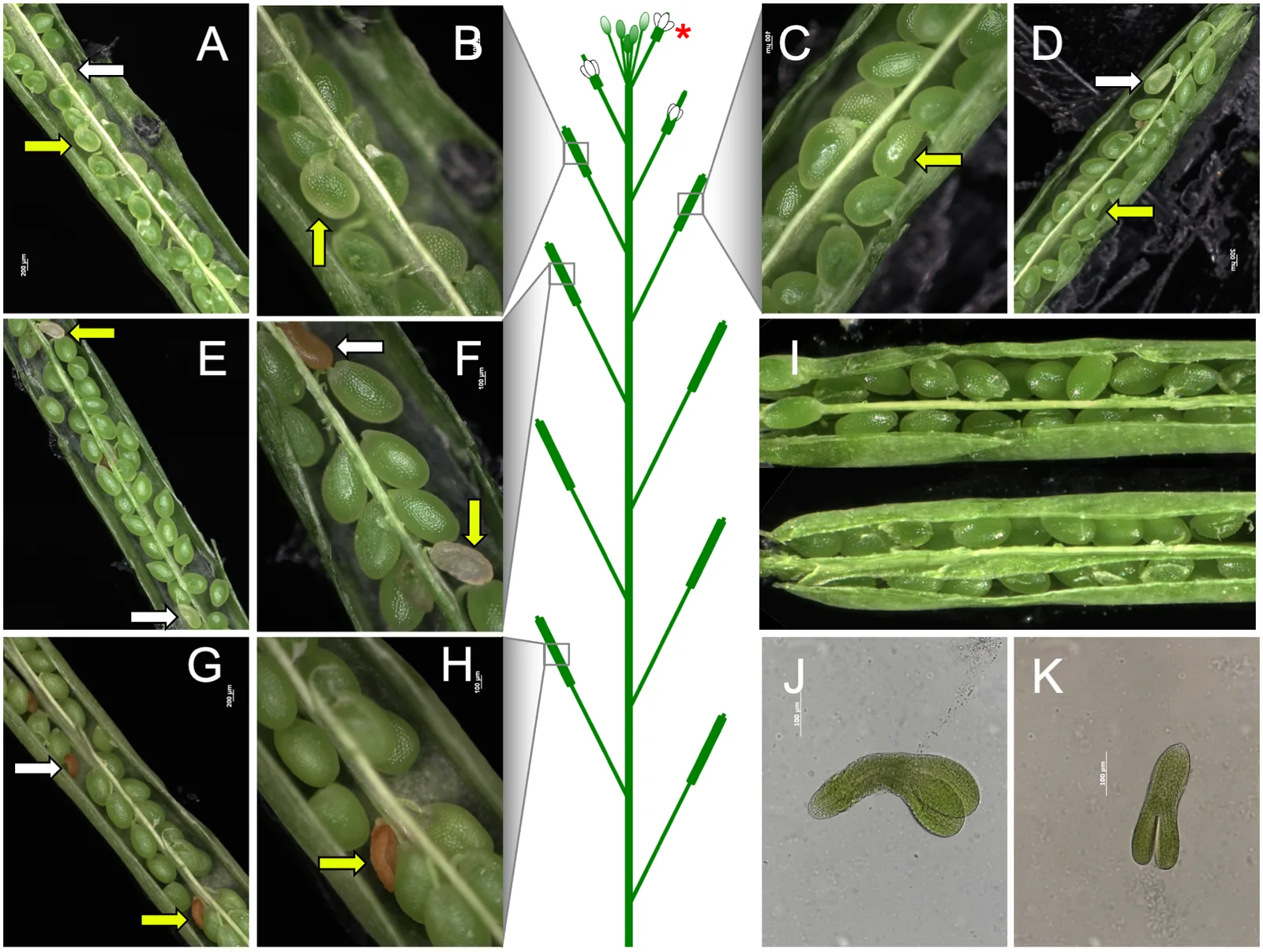
Seed development in the *aclb1;aclb2* double mutant. Developing seeds within dissected siliques at different stages of development from plants that have a *ACLB2-1/aclb2-1*; *ACLB1-1/aclb1–1* genotype **(A–H)** or from sibling wild type plants **(I)**. The schematic of the bolt in the center of the figure indicates the developmental stage of the silique, staged by the position of the siliques with respect to the youngest open flower (marked by a red asterisk): position 4 **(A, B)**, position 5 **(C, D)**, position 6 **(E, F)**, and position 10 **(G, H)**. Siliques from sibling wild type plants were imaged from position 6 **(I)**. Images were obtained by stereomicroscopy at either 3x magnification **(A, D, E, G, I)** or at 7x magnification **(B, C, F, H)**. White arrows identify developing seeds displaying an abnormal phenotype. Pairs of yellow arrows at each stage of development identify the identical seed that was imaged at different magnification. Images of embryos dissected from ovules within siliques at position 5 were from those showing the abnormal phenotype (i.e., *aclb1; aclb2* double mutant embryos) **(J)** and from wild type sibling plants **(K)**.

Embryos were dissected from the *aclb1; aclb2* double mutant ovules that display the abnormal phenotype prior to oval collapse (i.e., from siliques at position #5), and their appearance was compared to analogous embryos dissected from wild type sibling plants (c.f., **Figures 2J, K**). The embryos in the abnormal ovules were arrested at the torpedo stage of development (**Figure 2J**), whereas the corresponding wild type embryos were further advanced in their developmental program (**Figure 2K**). Therefore, we conclude that plants that are not expressing any ACLB subunit (i.e., *aclb1; aclb2* double mutants), which are incapable of expressing ACL enzymatic activity (Fatland et al., 2002), are not viable past the torpedo stage of embryo development.

Having established that complete loss of ACLB function is embryo-lethal, we noted that among the 280 viable progeny recovered from the self of double heterozygous plants (i.e., *ACLB2/aclb2-1; ACLB1/aclb1–1* genotype), 40 plants displayed a pronounced dwarf phenotype resembling the previously described “bonsai” morphology associated with the antisense *ACLA* plants (Fatland et al., 2005). Genotyping revealed that all 40 of these plants shared the genotype, *aclb1-1/aclb1-1; ACLB2/aclb2-1*, indicating that they carried only a single functional *ACLB* allele. This phenotype became evident early in development (14 days after imbibition) and persisted through rosette expansion and bolting (**Figure 3A**). At maturity, these plants reached 13 ± 2 cm in height and produced 32 ± 3 mg seed per plant (*n* = 40). By comparison, all other plants in this segregating family exhibited normal morphology throughout development. In particular, plants that lacked a functional *ACLB1* gene but retaining two wild type *ACLB2* alleles (i.e., *aclb1/aclb1; ACLB2/ACLB2*) reached a height of 25 ± 2 cm and produced 600 ± 85 mg seed per plant (*n* = 21) (**Figure 3A**). Thus, while a single wild type allele of *ACLB1* alone is sufficient to support viability, a single remaining *ACLB2* allele cannot sustain normal growth, demonstrating synthetic haploinsufficiency (or dosage effect) of the *ACLB2* allele to affect vegetative growth and development.

**Figure 3:**
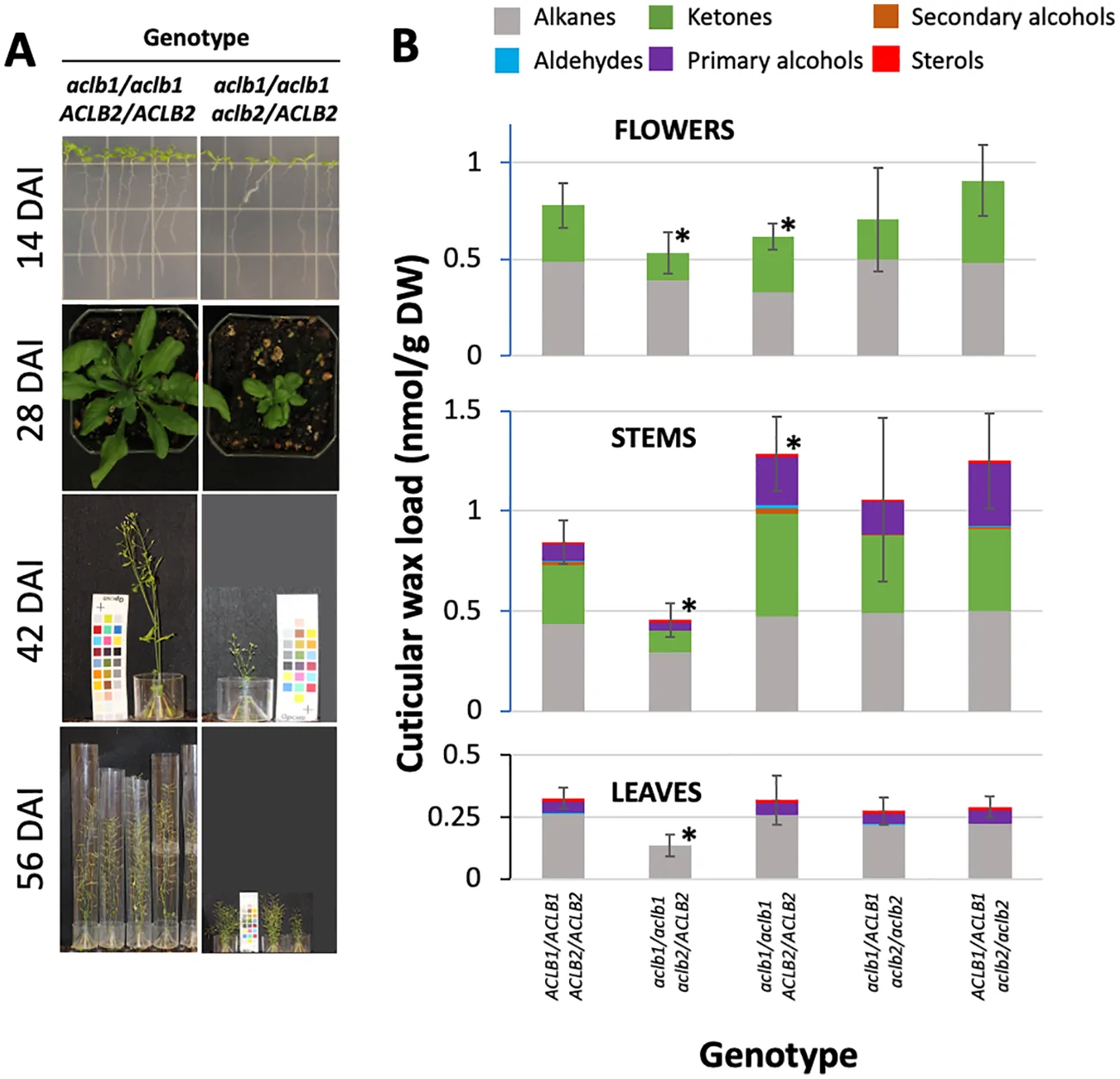
The effect of *aclb2* genetic dosage on growth and cuticular waxes. **(A)** Growth of plants that carried either two or one wild-type copies of the *ACLB2* gene but lacked a functional *ACLB1* gene. **(B)** Cuticular wax loads on flowers, stems and leaves of 18d-old seedlings of the indicated genotype. Average data ± standard deviation from 3–4 biological replicates, and each replicate was the pool of 3–6 seedlings. Asterisks (*) indicate statistically significant differences between mutant and wild type plants as determined by Student t-test (p<0.05).

### 3.5 Metabolic consequence of *acl* mutations

The functional redundancy among the three *ACLA* and two *ACLB* paralogs was explored at the biochemical level by evaluating the accumulation of phytochemicals whose biosynthesis are dependent on ACL enzymatic activity. Prior characterizations of antisense *acla* RNA plants that expressed reduced ACL activity (Fatland et al., 2005) established the crucial metabolic function of this enzyme in generating cytosolic acetyl-CoA, which can be converted to malonyl-CoA, a reaction catalyzed by the homomeric acetyl-CoA carboxylase (Roesler et al., 1994; Yanai et al., 1995). This malonyl-CoA pool is the source of the 2-carbon units used for the elongation of preexisting fatty acids, or is a substrate in the biosynthesis of a variety of different malonate-derived metabolites, collectively known as polyketides (e.g., anthocyanins). In this study therefore, we dissected this metabolic functionality using the viable genetic stocks that were missing specific ACLA or ACLB subunits, and quantified the accumulation of anthocyanins, VLCFAs (i.e., fatty acids with 20-carbon atoms and longer) and cuticular waxes, the latter being metabolically derived from VLCFAs.

Anthocyanin content was assessed in 18-d old seedlings, and because anthocyanin accumulation is known to be induced when plants are grown in elevated illumination conditions (Ma et al., 2021; Araguirang and Richter, 2022), these measurements were made with seedlings that were grown under either “normal” levels of illumination throughout the growth-period (photon flux density ~70-90 μmol*m^-2^*s^-1^), or after exposing the seedlings to elevated levels of illumination (photon flux density ~250-300 μmol*m^-2^*s^-1^) for the last 48 h of growth. Under normal illumination conditions, anthocyanin accumulation in single *acl* mutants was either decreased (~50% reduction in the two *acla3* mutants) or was indistinguishable from the wild type siblings (**Figure 4A**; **Supplementary Table S6**). The exception to this was the unexpected finding that in plants lacking a functional *ACLB2* gene, anthocyanin levels increased by ~4-fold.

**Figure 4:**
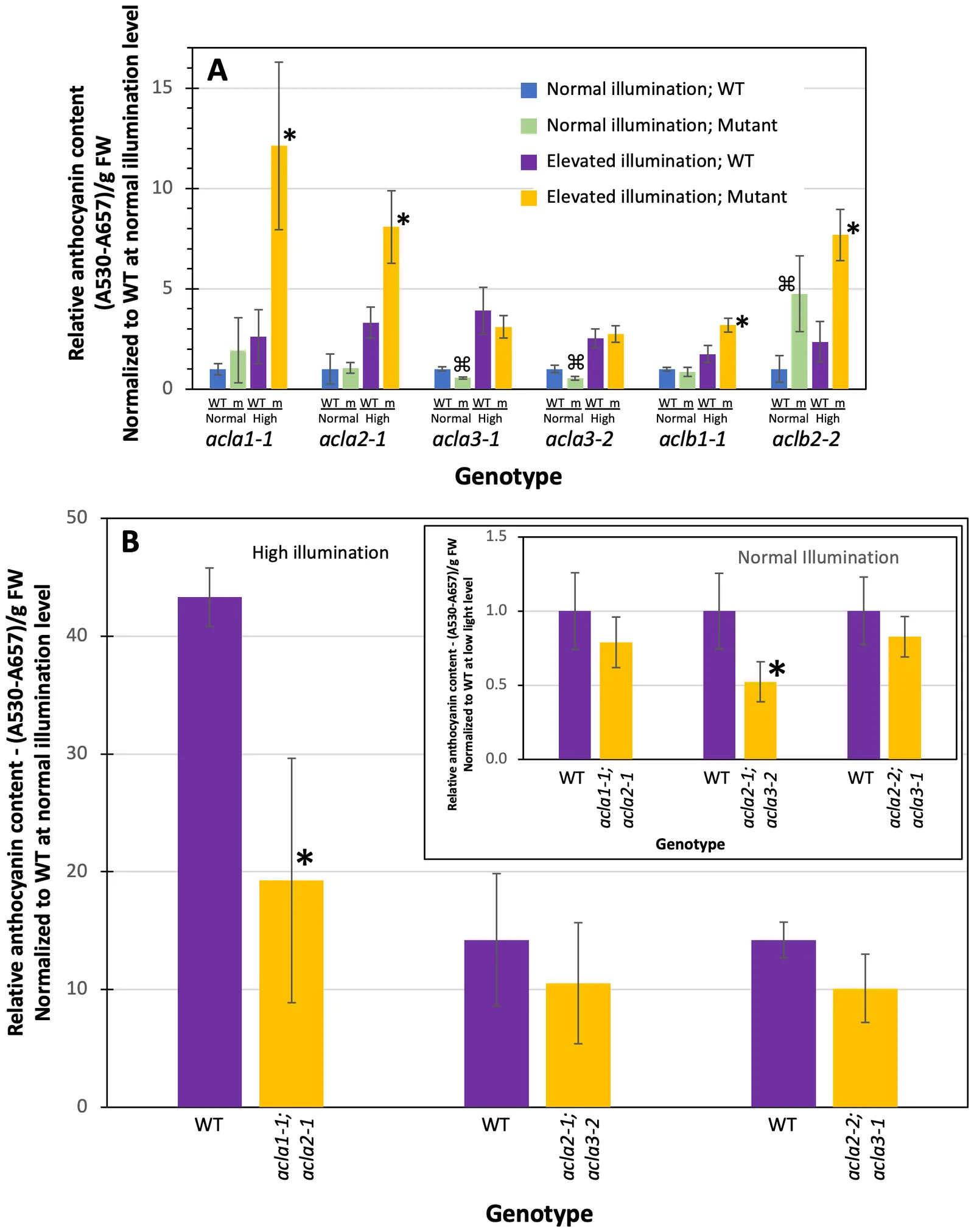
The effect of *acl* mutation on the accumulation of anthocyanins accumulation. **(A)** Anthocyanin content in 18-d old wild type (WT) or sibling mutant (m) seedling of the indicated *acl* allele. Plants were grown either under normal illumination conditions (Normal) or under high illumination intensity for the last 48 h of growth prior to harvesting (High). **(B)** Anthocyanin content in 18-d old wild type (WT) or sibling double mutant seedlings. Plants were grown either under normal illumination conditions (insert graph) or under high illumination intensity for the last 48 h of growth prior to harvesting. Average data ± standard deviation from 3–5 replicates, and each replicate was a pool of 3–6 seedlings. Asterisks (*) indicate statistical significance between mutant and wild type plants in the high illumination conditions (p<0.05), and ⌘ indicates statistical significance between mutant and wild type plants in the normal illumination conditions (p<0.05).

When wild type seedlings were exposed to high illumination levels, anthocyanin content increased by ~3-fold (**Figure 4A**; **Supplementary Table S6**). The effect of each of the *acl* mutations on this light-induced increase in anthocyanin levels varied among the different alleles. In seedlings lacking a functional *ACLA3* gene, the light-stimulated induction of anthocyanin was indistinguishable from the wild type seedlings. In the other 4 single mutant plants that lacked either *ACLA1*, *ACLA2*, *ACLB1* or *ACLB2*, anthocyanin content was hyper-induced by the high-light treatment, reaching ~4-fold higher levels than that obtained by the identical treatment of wild type siblings (**Figure 4A**; **Supplementary Table S6**).

**Figure 4B**, **Supplementary Table S6** show the results of analogous experiments conducted with the three *acla* double mutant lines. In seedlings grown in normal illumination levels, there were modest reductions in anthocyanin content (**Figure 4B**, insert; **Supplementary Table S6**). The high-light treatment caused a 15- to 40-fold increase in anthocyanin content of the wild type seedlings, but the *acla1; acla2* double mutant that carried only the functional *ACLA3* gene was unable to reach these high levels anthocyanin accumulation (**Figure 4B**, insert; **Supplementary Table S6**). Collectively, these experiments indicate that of the three *ACLA* paralogs, the *ACLA3* gene was primarily associated with generating the acetyl-CoA precursor needed for anthocyanin biosynthesis.

Fatty acid analyses focused on tissues that are known to accumulate relatively larger quantities of VLCFAs; these being seeds and roots. In seeds, VLCFAs are building blocks of the oil (i.e., triacylglycerols), which contains ~30% (mol/wt) of 20- and 22-carbon VLCFAs (O'Neill et al., 2003). Similarly, the membrane phospholipids of roots contain ~15% (mol/wt) of VLCFAs (Devaiah et al., 2006). Because fatty acids are most often covalently bound to more complex molecules, via ester, thioester, amide, or phosphoryl bonds, we first treated the biological materials with a strong base to hydrolyze these bonds and liberate free fatty acids, which were recovered and analyzed as methyl esters.

Seed fatty acid content varied among the genotype groups (one-way ANOVA, P = 0.0016); however, Tukey’s HSD multiple-comparison test identified no significant differences between any mutant and its corresponding wild-type sibling control (adjusted P > 0.05; **Figure 5A**; **Supplementary Table S7**).

**Figure 5:**
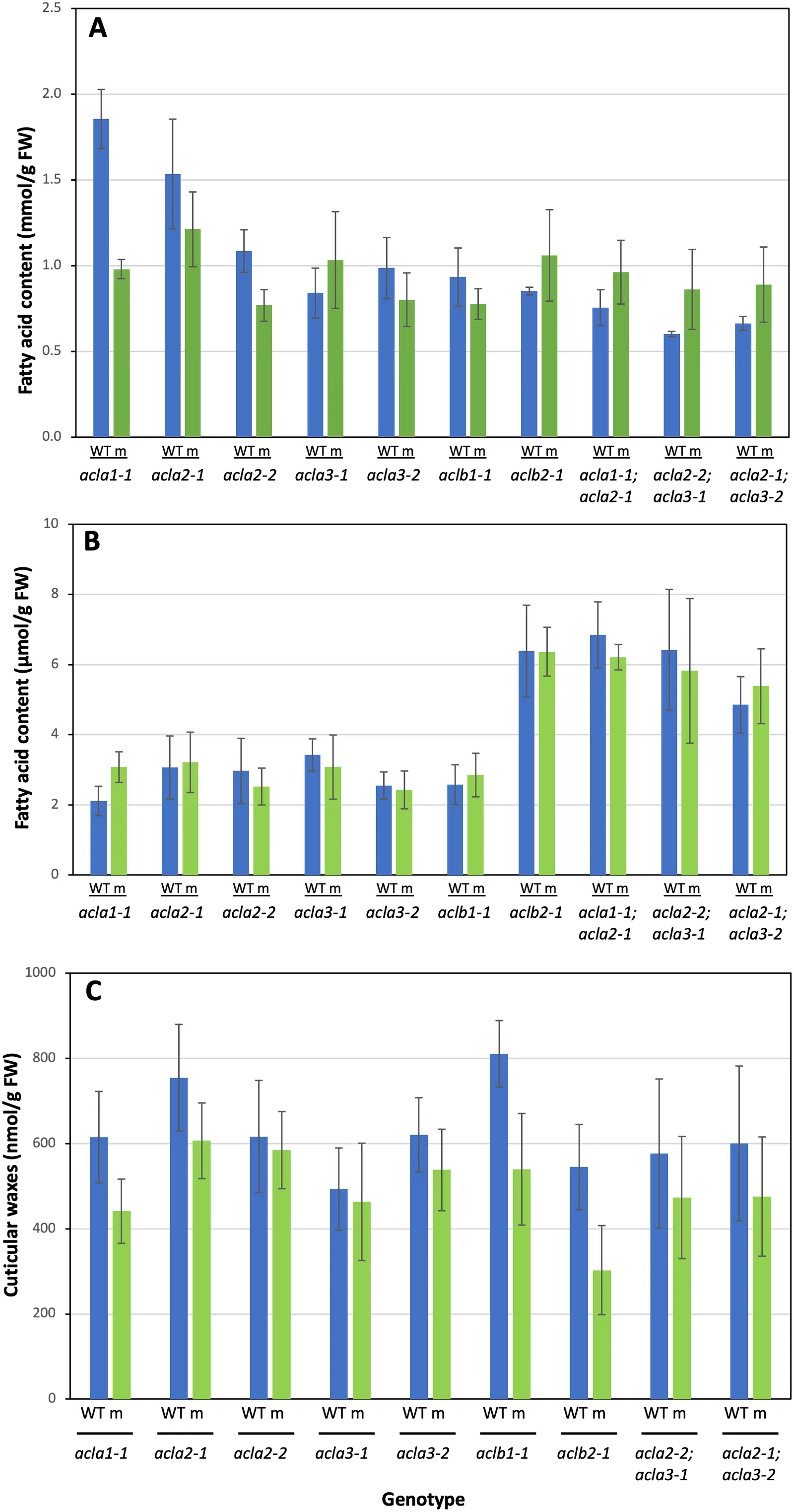
The effect of *acl* mutations on the accumulation of fatty acids and cuticular waxes. Fatty acid content in seeds **(A)**, seedling roots **(B)** and cuticular waxes on seedlings **(C)** of sibling wild type (WT) and single or double *acl* mutants (m). Seeds were collected from dry, fully mature plants, roots and cuticular waxes were analyzed from 18d-old seedlings. Data are presented as mean ± SD from 3–4 biological replicates, with each biological replicate consisting of a pool of 3–6 seedlings. Statistical significance was evaluated separately for each panel by one-way ANOVA followed by Tukey’s HSD multiple-comparison test. No mutant differed significantly from its corresponding wild-type sibling control after correction for multiple comparisons (adjusted P > 0.05).

Similarly, no mutant differed significantly from its corresponding wild-type control in root fatty acid content following one-way ANOVA and Tukey’s HSD multiple-comparison analysis (**Figure 5B**; **Supplementary Table S8**). Regardless, in all these lines the VLCFAs accounted for ~30 mol% of the seed (**Supplementary Figure S8A**) or root (**Supplementary Figure S8B**) fatty acids, and this was unaffected by any of the single or double mutants. These findings indicate that the supply of cytosolic acetyl-CoA does not limit the generation of VLCFAs.

We also analyzed the solvent extractable cuticular lipids that accumulate on the aerial surfaces of 18 d-old seedlings, commonly known as cuticular waxes. These are metabolically derived from the cytosolic pool of acetyl-CoA, using VLC fatty acyl-CoA intermediates (Baales et al., 2021). In agreement with prior analyses (Jenks et al., 1995), these cuticular waxes included alkanes (~50 mol %), primary and secondary alcohols (~20 mol %), VLCFAs (~5 mol%) and ketones (~2 mol%). The other major components of the detected cuticular waxes (~20 mol%) were sterols (e.g., sitosterol, cholesterol, and dihydrobrassicasterol) (Jetter et al., 2006), which are not metabolically derived from VLC fatty acyl-CoA, but are synthesized from cytosolic acetyl-CoA, via acetoacetyl-CoA and mevalonate-intermediates (Bach et al., 1999; Jin et al., 2012). Cuticular wax accumulation varied among the genotype groups (one-way ANOVA, P = 0.0011), but Tukey’s HSD multiple-comparison test detected no significant difference between any mutant and its corresponding wild-type sibling control (adjusted P > 0.05; **Figure 5C**; **Supplementary Table S9**). Although cuticular wax levels tended to be lower in several mutants, including *aclb1–1* and *aclb2-1*, these differences were not significant after correction for multiple comparisons.

### 3.6 Molecular characterization of synthetic haploinsufficiency of the *ACLB2* allele

Consistent with the quantitative requirement for *ACLB2* activity, cuticular wax loads on flowers, stems and leaves were reduced by approximately 50% in plants that carried only a single wild type *ACLB2* allele (i.e., genotype: *aclb1-1/aclb1-1; ACLB2/aclb2-1*), whereas all other genotypic classes that carried two wild type *ACLB2* alleles accumulated waxes at levels comparable to wild type (**Figure 3B**). These findings indicate that total ACLB activity is not only essential for embryo viability but is also rate-limiting for post-embryonic growth and cuticular lipid biosynthesis.

A broader perspective of the consequence of varying the *ACLB2* gene dosage was obtained by sequencing the transcriptome of seedlings of five different genotypes at two different stages of development (i.e., at 18- and 24-DAI). These seedlings carried either two wild type copies of *ACLB1* and *ACLB2* genes (i.e., wild type seedlings), or one or two wild type copies of either *ACLB1* or *ACLB2* genes in coupling with two homozygous mutant copies of either *aclb2* or *aclb1*, respectively. Thus, these mutant seedling carried either two, one or no wild type copies of the *ACLB2* or *ACLB1* genes.

Orthogonal linear transformation of the transcriptomic data by Principal Component Analysis indicated that age of the seedlings was the major difference among the transcriptomes (i.e., Principal Component 1; **Figure 6A**). Moreover, these analyses indicate that with the exception of the *aclb1/aclb1; aclb2/ACLB2* seedlings at 24 DAI, the transcriptomes of all other genotypes, at both 18- and 24-DAI, were indistinguishable from the wild type (**Figure 6A**). These results are consistent with the finding that apart from the *aclb1/aclb1; aclb2/ACLB2* seedlings, all other genotype combinations showed morphological (**Figure 3A**) and cuticular wax biochemical phenotypes (**Figure 3B**) that were indistinguishable from the WT seedlings.

**Figure 6:**
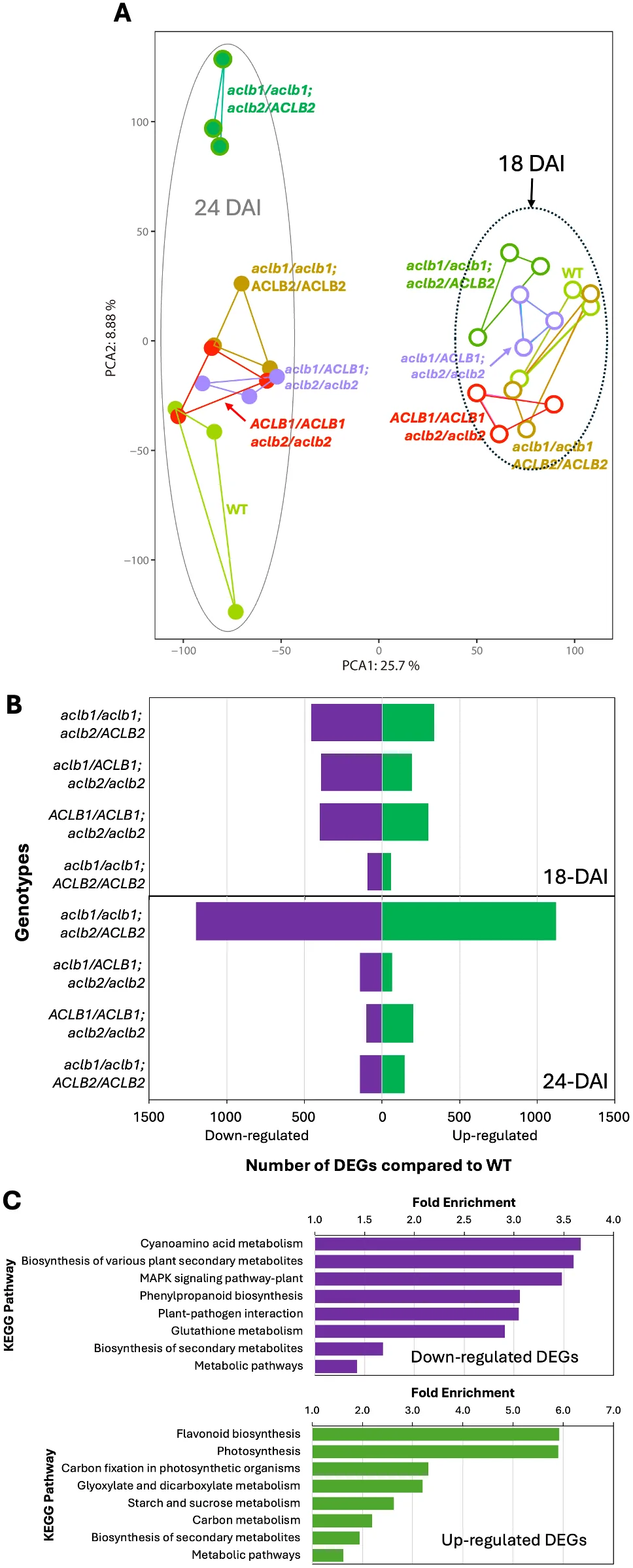
Transcriptomes of *aclb* mutants. **(A)** PCA visualization of transcriptomes of seedlings at 18-DAI and 24-DAI of the indicated combinations of *aclb1* and *aclb2* mutants. **(B)** Number of differentially expressed genes (DEGs) of different combinations of *aclb1* and *aclb2* mutants as compared to wild-type seedlings at 18-DAI and 24-DAI. **(C)** KEGG pathway enrichment analysis with the list of down-regulated or up-regulated DEGs of *aclb1/aclb1; aclb2/ACLB2* genotype versus wild type 24-DAI seedlings.

The transcriptomes from each of the mutant seedlings were compared to the transcriptome of the wild type seedlings to identify differentially expressed genes (DEGs) (**Supplementary Table S10, S11**). Approximately 2, 500 DEGs were identified, and they were nearly equally divided between those that are up-regulated and those that are down-regulated, relative to the wild type (**Figure 6B**). Notably, the plants with only a single wild type *ACLB2* allele (i.e., *aclb1/aclb1; aclb2/ACLB2* genotype) exhibited the highest number of DEGs, totaling approximately 2, 400 genes at 24 DAI (**Figure 6B**), which correlates with the observed seedling phenotype (**Figure 3A**). In contrast, all other mutant seedling samples, including those with two wild type *ACLB2* alleles (i.e., *aclb1/aclb1; ACLB2/ACLB2* genotype) showed fewer than ~800 DEGs each (**Figure 6B**).

Insights on the metabolic functionalities that were affect by the *ACLB2* gene dosage effect was gained by interrogating the ShinyGO analytical platform (Ge et al., 2019) with the up-regulated and down-regulated DEGs of the 24-DAI seedlings, which carried only a single functional copy of *ACLB2*. The list of down-regulated DEGs (1199 genes) identified KEGG stress pathways associated with secondary metabolism (e.g., phenylpropanoid metabolism, glutathione metabolism) (**Figure 6C**). Whereas, the list of up-regulated DEGs (1122 genes) identified KEGG pathways associated with central carbon metabolism (e.g., photosynthesis, carbon fixation, sugar metabolism, glyoxylate and dicarboxylate metabolism) (**Figure 6C**).

## 4 Discussion

Prior transgenic studies that down-regulated ACL expression with antisense-RNA technology (Fatland et al., 2005) or up-regulated ACL expression (Xing et al., 2014) have established that ACL-generates the cytosolic acetyl-CoA pool required for the normal growth of seedlings, and that no other source of acetyl-CoA can substitute (Fatland et al., 2005). In *Arabidopsis*, ACL is encoded by five genes, three encoding the ACLA subunit and two encoding the ACLB subunit (Fatland et al., 2002). The current study genetically dissected the role of each *ACL* subunit-gene in determining the physiological functionality of ACL. Specifically, we characterized plants carrying a combination of *acla-* or *aclb*-mutant null alleles.

### 4.1 Molecular genetics of *ACLA* genes

The five genetic lines that carried individual mutant *acla* alleles were phenotypically silent, presenting a normal morphological appearance, and were inherited in Mendelian ratios consistent for recessive, non-lethal alleles. Thus, each of the three *ACLA* genes appear to be functionally redundant. In the case of the *ACLA1* and *ACLA3* genes, their functional redundancy was also confirmed by the characterization of double mutant plants that expressed only one these *ACLA* genes. The same could not be determined for the *ACLA2* gene, because an *acla1, acla3* double mutant plant could not be generated due the proximity of these two genes on chromosome 1 of *Arabidopsis*.

The metabolic consequence of these single and double mutants was assessed by analyzing metabolites that are derived from the cytosolic acetyl-CoA pool (i.e., VLCFAs, cuticular waxes and anthocyanins). As would be expected for functionally redundant genes, these mutations did not significantly affect the accumulation of VLCFAs or cuticular waxes. We infer therefore that residual ACL enzymatic activity in these viable mutants is sufficient to meet the metabolic demand for VLCFA biosynthesis. The only consistent biochemical change noted in these mutants was the increased accumulation of anthocyanins. Although this finding is counter intuitive with the expected decrease in the availability of the acetyl-CoA precursor of anthocyanins, it is consistent with the prior finding of increased anthocyanin content in the antisense *ACLA* plants (Fatland et al., 2005). Thus, despite the reduced availability of cytosolic acetyl-CoA supply due to *acl* mutations, anthocyanin levels increase. This finding may indicate a prioritization of acetyl-CoA metabolism towards anthocyanin biosynthesis, possibly as a response to a stress situation as a consequence of the non-functionality of the full complement of *ACLA* genes.

### 4.2 Molecular genetics of *ACLB* genes

Analogously, four different null alleles were used to explore the functional redundancy between the *ACLB1* and *ACLB2* genes. Because there are only two *ACLB* paralogs in the *Arabidopsis* genome, which are genetically not linked, it was possible to evaluate the essentiality of the ACLB protein, and thereby the ACL enzyme. A combination of two observations indicate that ACL catalytic function is essential during embryo development. Specifically, *aclb1; aclb2* double mutant plants were not recoverable among the progeny of double heterozygous plants (i.e., *aclb1/ACLB1; aclb2/ACLB2* genotype), even though all other anticipated genotypes were recoverable at the expected Mendelian segregation ratio. Additionally, siliques developing on these double heterozygous plants contained aborted seeds at a rate of ~6%, which matches the expected proportion of progeny with *aclb1; aclb2* double mutant genotype. Moreover, these aborted seeds contained embryos that had not developed beyond the torpedo stage of development. Therefore, we conclude that the lack of ACL activity becomes essential as embryos transition through the torpedo stage of development.

### 4.3 Synthetic haploinsufficiency of the *ACLB2* allele

An unexpected finding revealed by the analysis of the progeny of the plants with the *aclb1/ACLB1; aclb2/ACLB2* genotype, was the observation that the *ACLB1* and *ACLB2* genes are not fully functionally redundant during vegetative growth. This conclusion is based on the observation that plants that carry only a single wild-type *ACLB2* allele (i.e., *aclb1/aclb1; aclb2/ACLB2* genotype) show an aberrant growth phenotype, whereas plants that carry only a single wild-type *ACLB1* allele (i.e., *aclb1/ACLB1; aclb2/aclb2* genotype) are indistinguishable from the wild-type; indeed all other genotypes recovered from the progeny of the double heterozygous plants presented a wild-type growth morphology. Thus, in the absence of a functional *ACLB1* gene, the genetic functionality of the *ACLB2* gene is haploinsufficient. Namely, whereas plants that carry two functional copies of *ACLB2* (i.e., *aclb1/aclb1; ACLB2/ACLB2* genotype) show a wild-type growth morphology, plants with only one functional copy of *ACLB2* (i.e., *aclb1/aclb1; aclb2/ACLB2* genotype) exhibit a growth deficiency phenotype. This phenotype is analogous to the “bonsai” phenotype previously described with plants that have down-regulated ACL expression by the use of an antisense-RNA transgene (Fatland et al., 2005).

Molecular phenotypes associated with the synthetic haploinsufficiency of the *ACLB2* gene were interpreted in the context of the metabolic function of the ACL enzyme; the generation of cytosolic acetyl-CoA. Specifically, cuticular wax loads on the three tissues examined (i.e., flowers, stems and leaves) showed reduction to ~50% of wild-type levels in parallel to the dosage of the *ACLB2* gene. More broadly, the transcriptomes of seedlings were remarkably unaffected by this gene dosage affect until later in growth (i.e., at 24 DAI versus 18 DAI), and even then, <10% of the transcribed genome was affected; the affected genes appeared to be associated with stress responses.

Haploinsufficiency occurs in eukaryotes from yeast to humans (Johnson et al., 2019). Initially the phenomenon was characterized in gene-by-gene analyses; for example in *Arabidopsis* it was characterized with *ERECTA*-family of genes: *ERL2* gene demonstrates synthetic haploinsufficiency in maintaining female fertility in the absence of the *ER* and *ERL1* genes (Pillitteri et al., 2007). In humans, a common genetic disorder often caused by haploinsufficiency of the *Low-Density Lipoprotein Receptor* gene is associated with elevated levels of serum cholesterol and a high risk of premature atherosclerosis (Freiberger, 2026). In yeast, cytoskeletal components such as actin (*ACT1*) (Drubin et al., 1993), tubulin (*TUB1*) (Schatz et al., 1986), and components of the spindle pole body (*NDC1*) (Chial et al., 1999) are haploinsufficient. A systematic study with the model organism, *S. cerevisiae*, found that 3% of the genome (184 genes) showed haploinsufficiency, the majority of which (98 genes) were essential (i.e., mutant homozygotes were not viable), and the other 86 genes showed growth defects in the homozygous mutant state (Deutschbauer et al., 2005). These yeast haploinsufficient genes are overwhelmingly involved in core metabolic processes, carried out by molecular complexes, as we have found for *Arabidopsis* ACL.

## Data Availability

The raw datasets generated for this study can be found in the **Supplementary Data Files**, and machine readable files versions of these datasets can be found at the Iowa State University DataShare site: https://doi.org/10.25380/iastate.33158366. Raw RNA-Seq data used in this paper is available from the NCBI Sequence Read Archive under accession number PRJNA1495842.

## In memoriam

This paper is dedicated to the late Dr. Ann Perera who provided insightful and supportive leadership at the W.M. Keck Metabolomics Research Laboratory, Iowa State University.
