# Supplementary material for: Functional redundancy and synthetic haploinsufficiency among Arabidopsis ATP-citrate lyase genes
Source: Front Plant Sci. 2026 Oct 9;17:1961717. doi: 10.3389/fpls.2026.1961717 (PMC13690957; doi:10.3389/fpls.2026.1961717)
Supplement: Supplementary file 1 [file DataSheet1.pdf]

Supplementary Material

1 Supplementary Figures

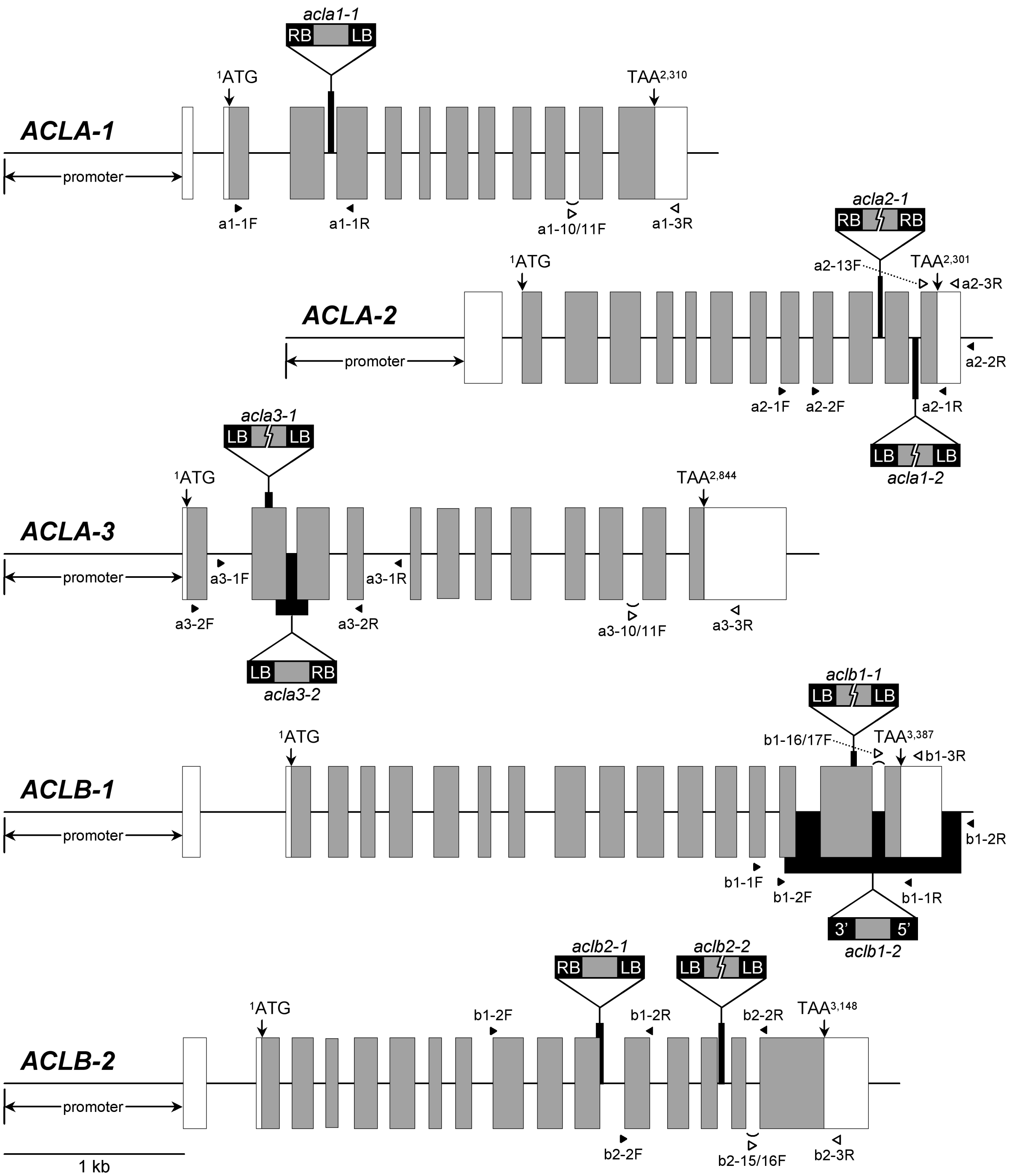

Supplemental Figure S1. Molecular structure of each *acl* gene and mutant alleles. The position of each insertion was determined by sequencing the DNA that flanks both sides of the insertion element (T-DNA or the *Ds* transposable element) that caused each mutation. These flanking DNA segments were isolated by PCR amplification with primer pairs that that were specific for each *ACL* gene sequence and the insertion element (Supplemental Table S1). The *acla1-1* (SALK_021437) allele was obtained from the Salk Institute Genomic Analysis Laboratory (SIGnAL) through the Arabidopsis Biological Resource Center (ABRC) at The Ohio State University (Alonso et al., 2003). The T-DNA insertion in the *acla1-1* allele is located in Intron 3, and is coupled with a 29-bp deletion. Two *acla2* mutant alleles were characterized. The *acla2-1* (FLAG_CFN21) was obtained from Versailles collection of the French National Institute for Agronomic Research (INRA) (Samson et al., 2002). The *acla2-1* mutation is caused by a T-DNA insertion that is located in Intron 11, coupled with a 7-bp deletion. The *acla2-2* allele (GABI_187H03) was obtained from GABI-Kat collection of the Genome Analysis of the Plant Biological System project (GABI) through the Nottingham Arabidopsis Stock Centre (Rosso et al., 2003). The *acla2-2* mutation is caused by a T-DNA insertion located in Intron 12 and is coupled with a 30-bp deletion. Two *acla3* mutant alleles were characterized. Both *acla3-1* (SALK_120573) and *acla3-2* (SALK_067448) were obtained from the SIGnAL collection. The *acla3-1* mutation is caused by a T-DNA insertion that is located in Exon 2 and is coupled with a 39-bp deletion. The *acla3-2* mutation is caused by a T-DNA insertion that is coupled with a 160-bp deletion, which begins in Exon 2 and ends in Exon 3, thereby completely eliminating Intron 2. Two *aclb1* mutant alleles were characterized. The *aclb1-1* (GABI_895F02) allele was obtained from the GABI-Kat collection, and is caused by a T-DNA insertion in Exon 16, with an associated 25-bp deletion. The *aclb1-2* (CS100148) allele was obtained from the IMA Ds transposon insertion collection through ABRC (Parinov et al., 1999). The *aclb1-2* mutation is associated with a 910-bp deletion, which begins in Exon 15 and continues through the 3’-UTR, thus eliminating the remainder of the *ACLB1* genomic sequence. Two *aclb2* mutant alleles were also characterized. The *aclb2-1* allele (FLAG_EIC213) was obtained from the Versailles collection, and it’s caused by a T-DNA insertion that is associated with a 39-bp deletion that reaches from Exon 11 into Intron 11. The *aclb2-2* allele (GABI_540H12) was obtained from the GABI-Kat collection, and is caused by a T-DNA insertion within Intron 14, and is associated with a 23-bp deletion.

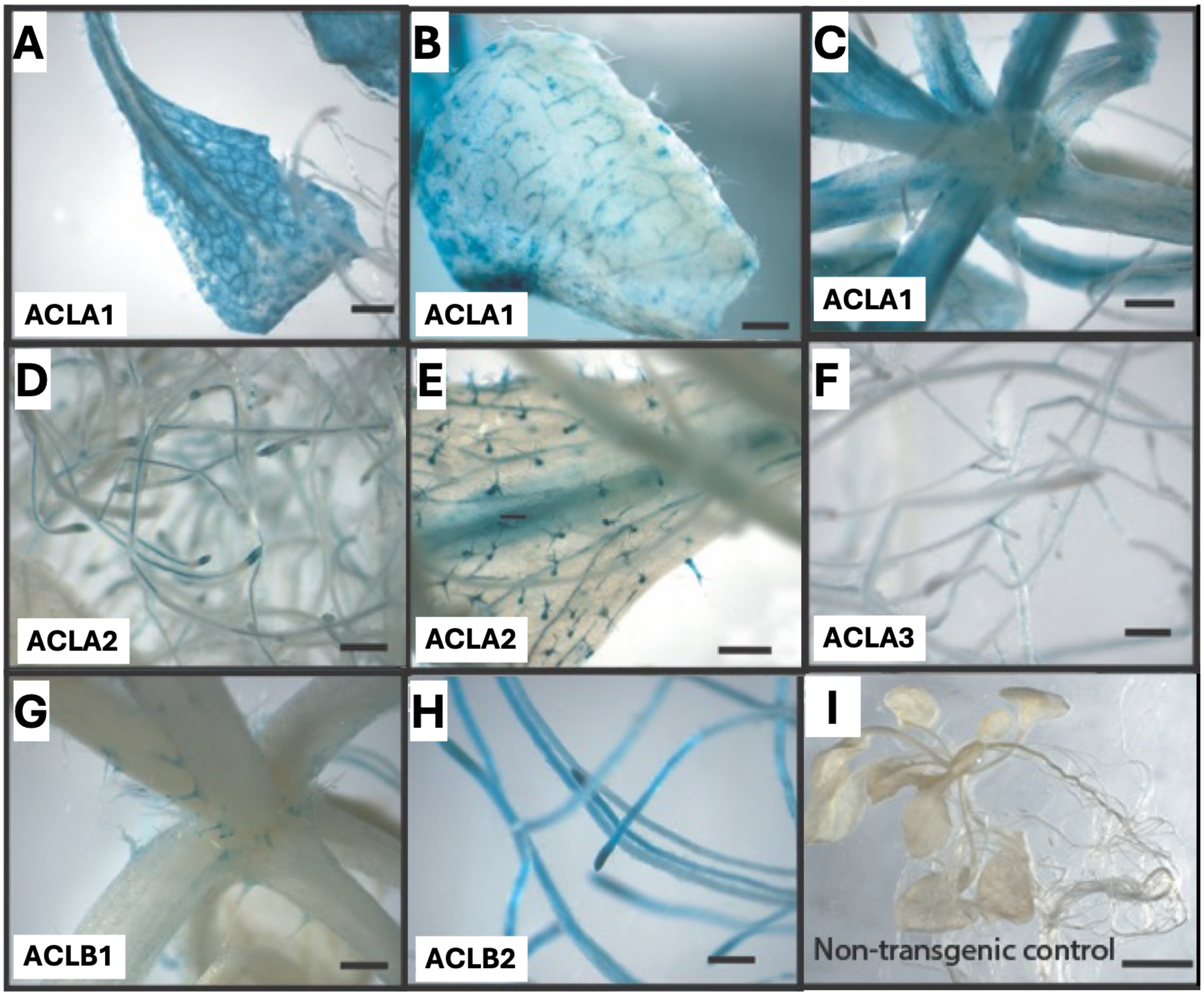

Supplemental Figure S2. Expression of ACL genes visualized by histochemical localization of *ACL* promoter-mediated GUS expression in 18-d old *Arabidopsis* seedlings. GUS activity was visualized by infiltrating the seedlings with 5-bromo-4-chloro-3-indolyl-b-D-glucoronic acid and incubating for 24 h. *ACLA1:GUS* rosette leaf (A), cotyledon (B), and shoot apical meristem (C); *ACLA2:GUS* roots (D), rosette leaf (E); *ACLA3:GUS* roots (F); *ACLB1:GUS* shoot apical meristem (G); and *ACLB2:GUS* roots (H). GUS expression was undetectable in non-transgenic seedlings (I). Scale-bars = 5 mm.

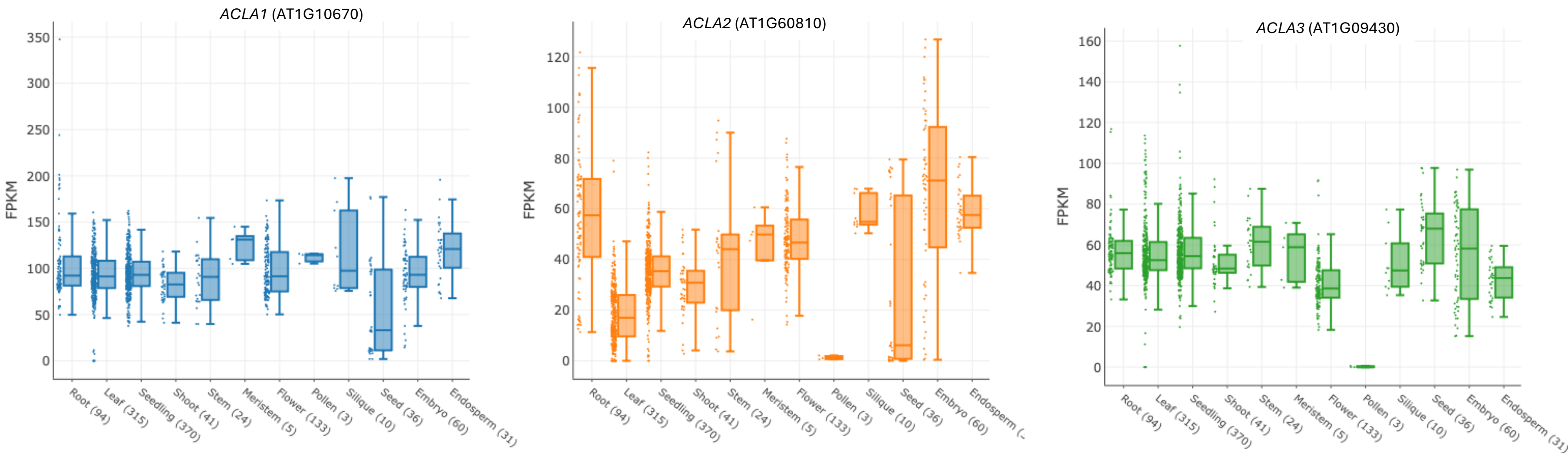

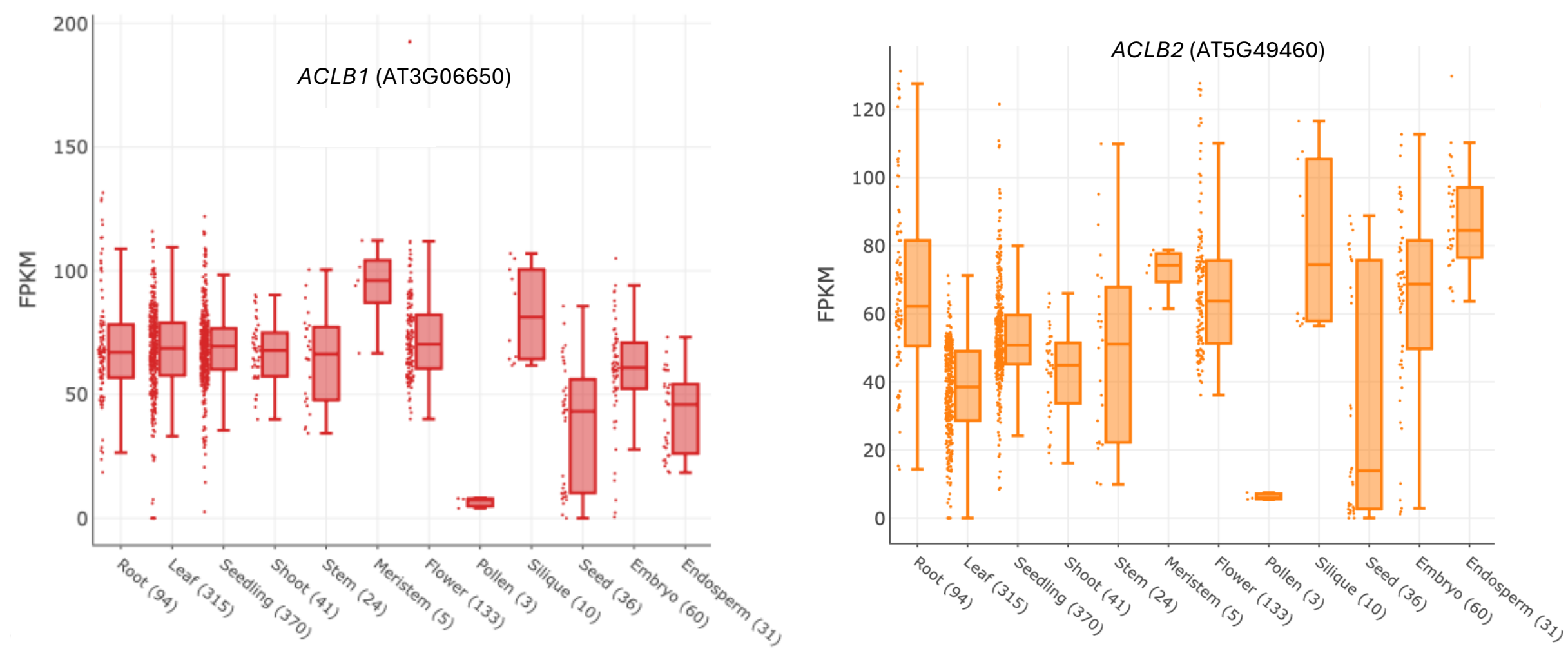

Supplemental Figure S3. Expression of *ACL* genes inferred from publicly available RNA-seq database (Zhang et al., 2020) (https://plantrnadb.com/athrdb/)

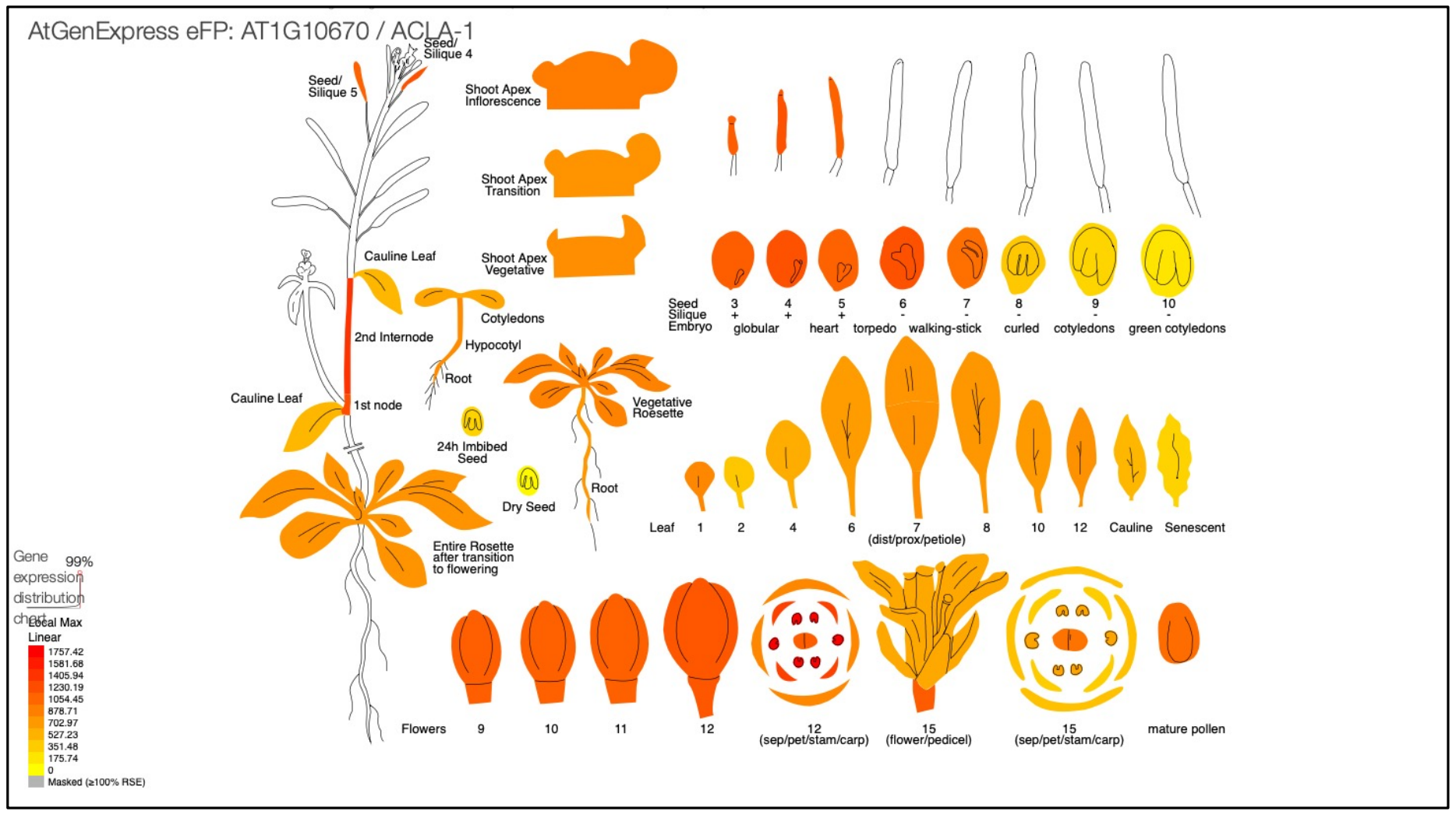

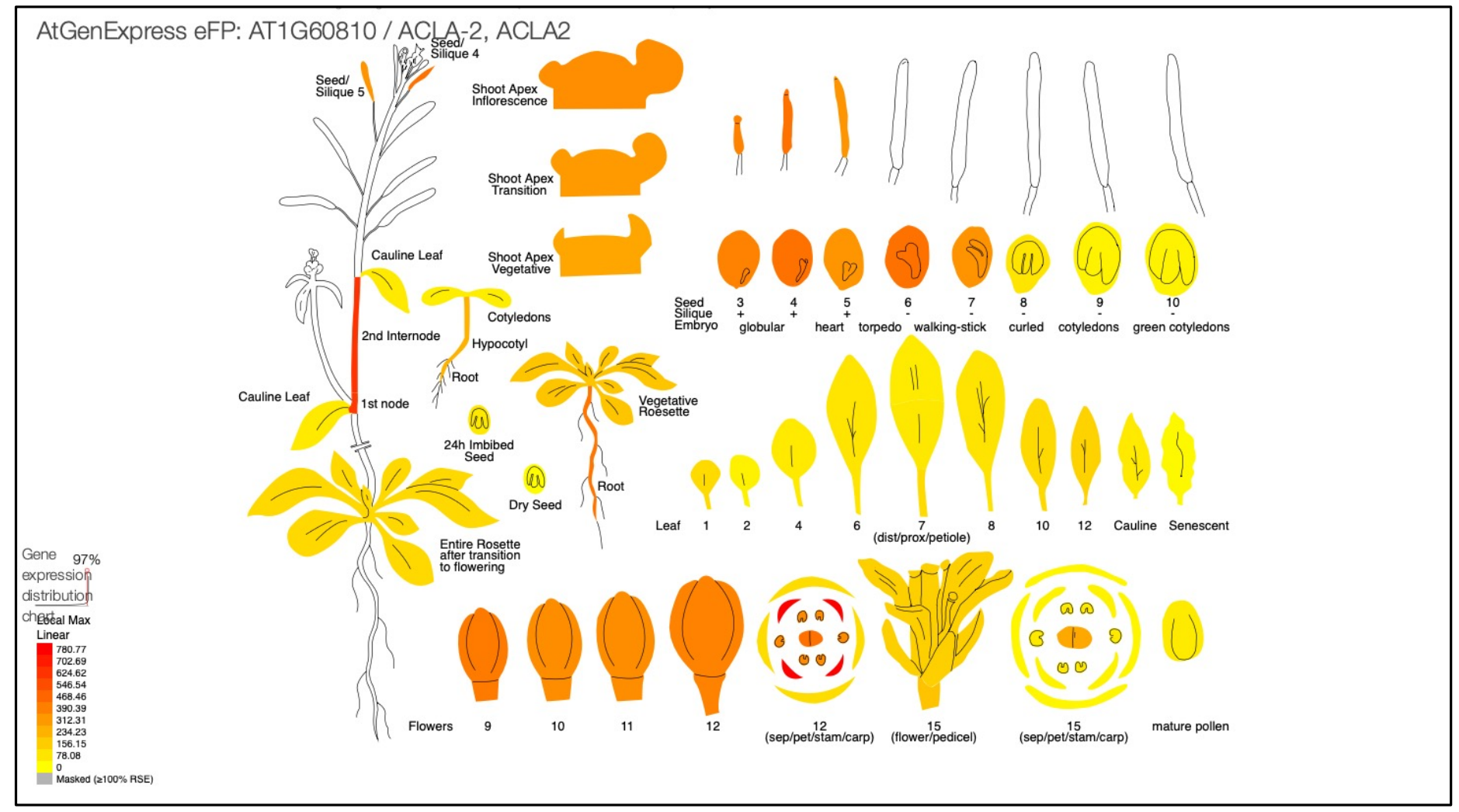

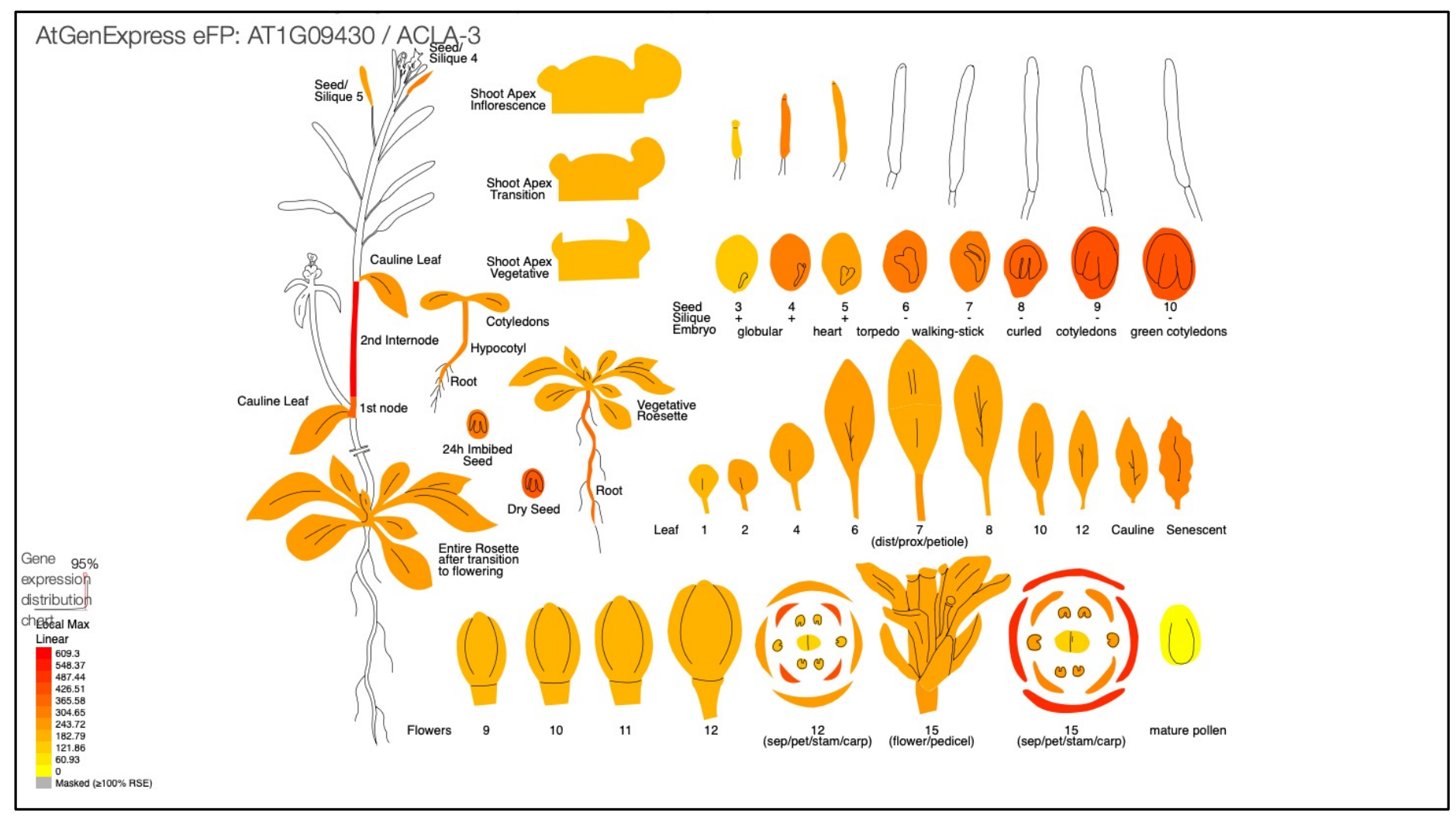

Supplemental Figure S4. Expression of the 3 *ACLA* genes visualized at the Arabidopsis eFP Browser (Winter et al., 2007).

ACLA2 expression relative to *ACLA1*

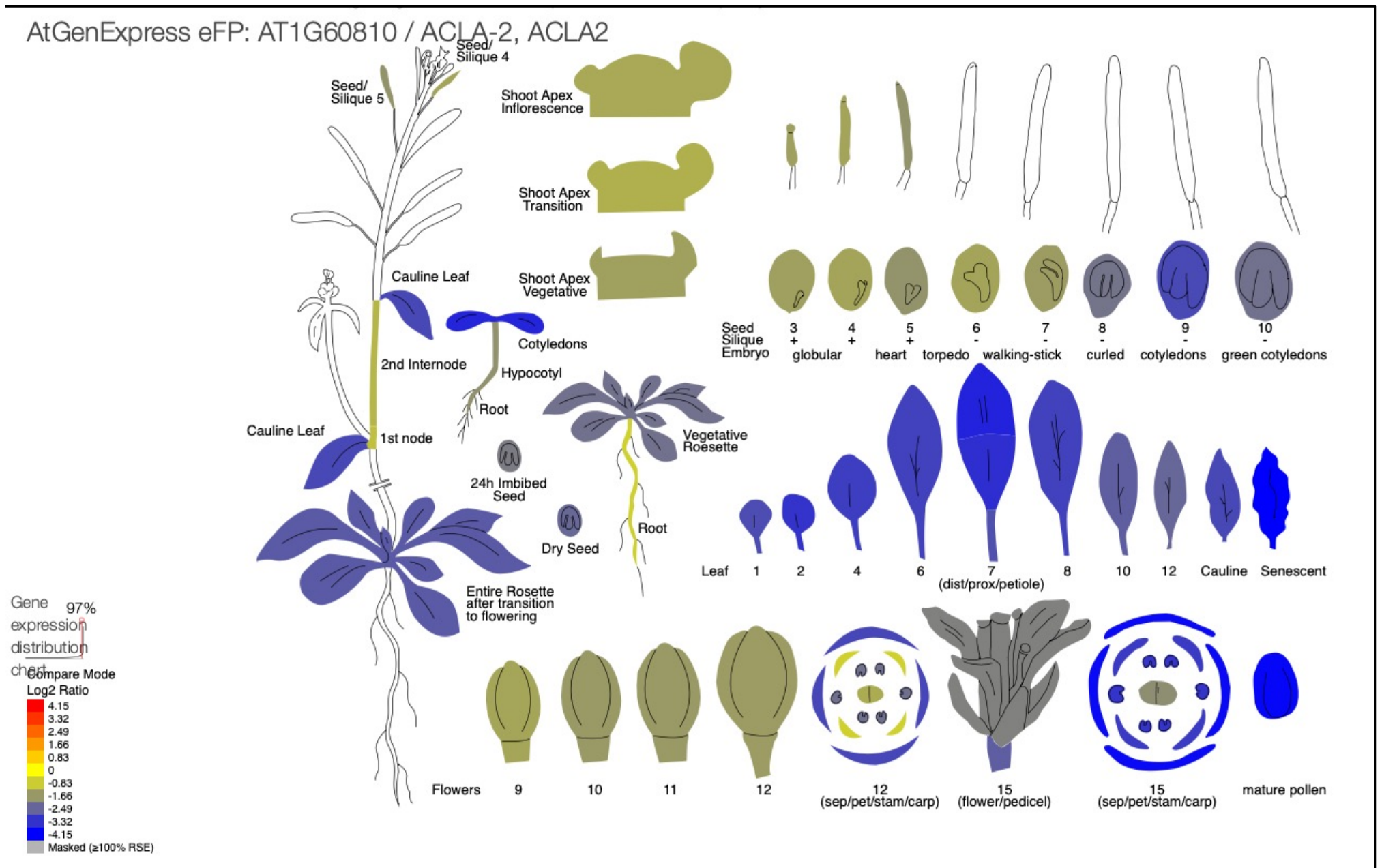

ACLA3 expression relative to *ACLA1*

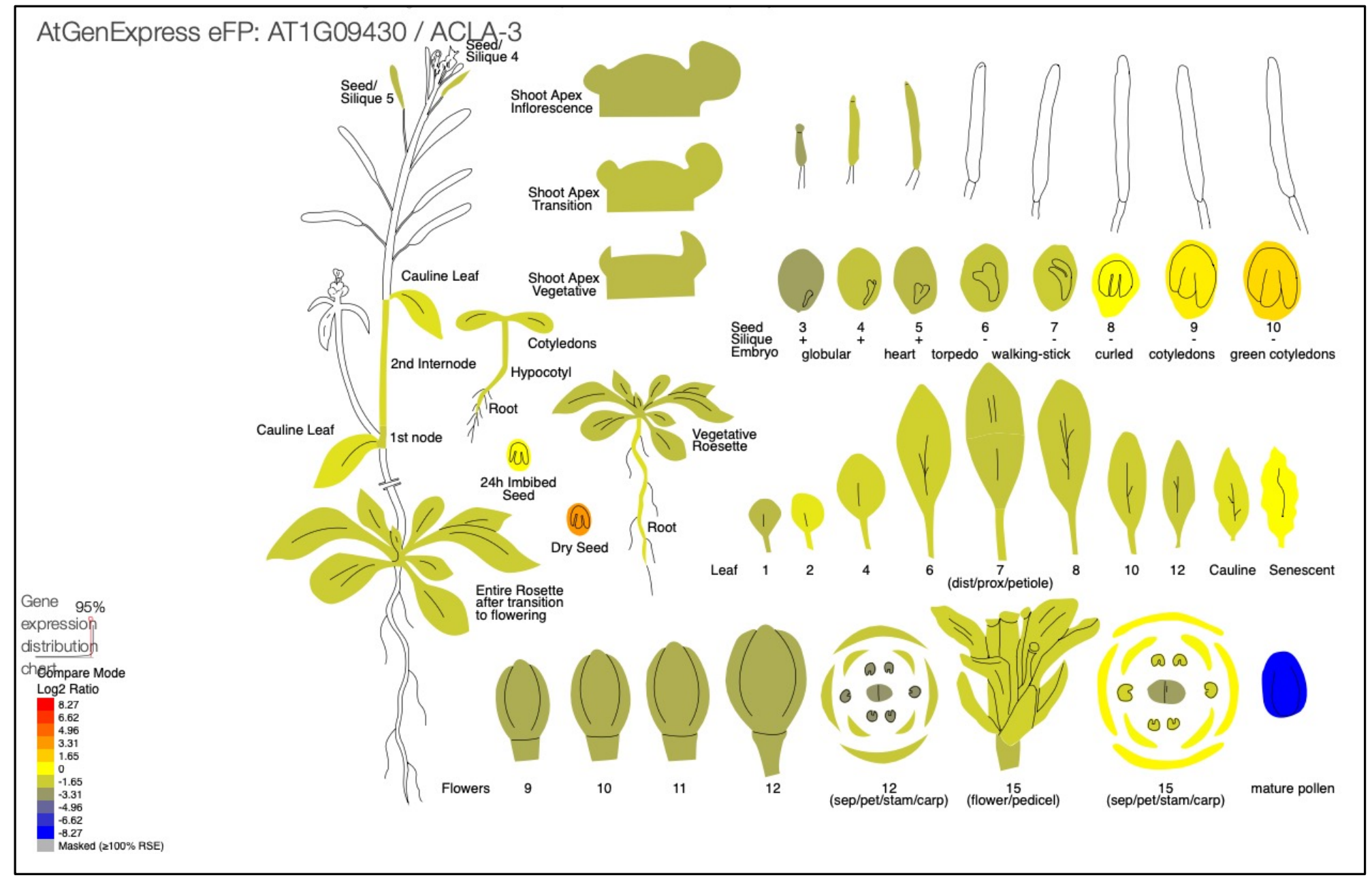

Supplemental Figure S5. Expression of *ACLA2* and *ACLA3* relative to the expression of *ACLA1*. Expression data were visualized at the Arabidopsis eFP Browser (Winter et al., 2007).

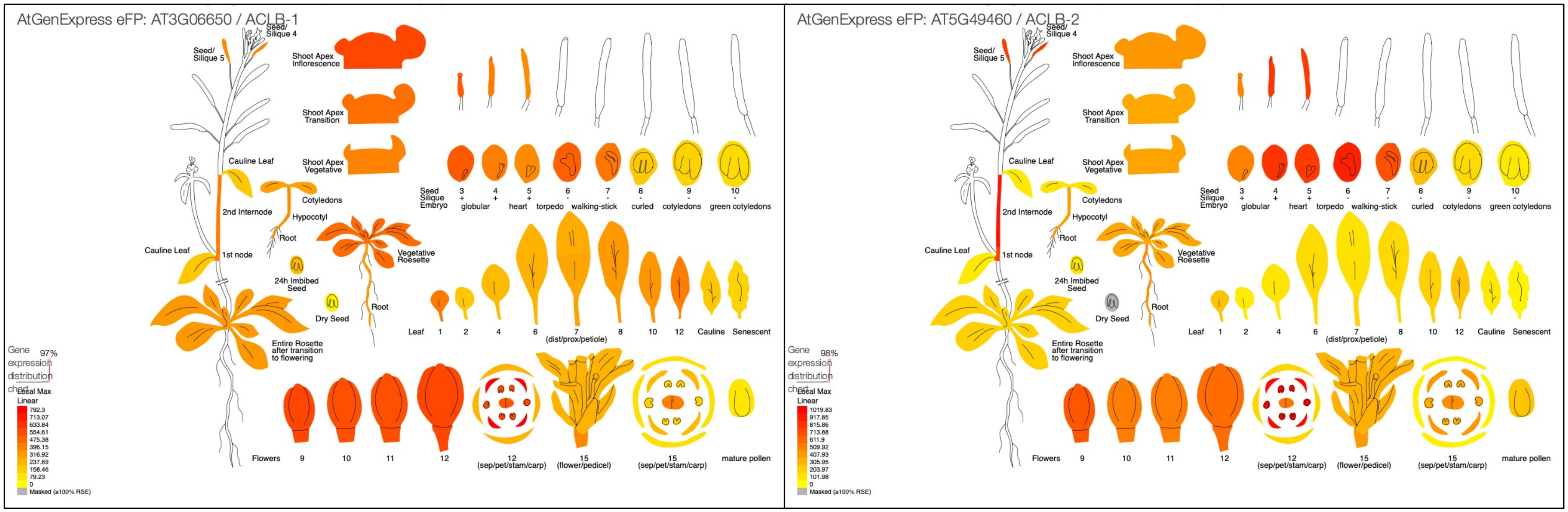

Supplemental Figure S6. Expression of the two *ACLB* genes visualized at the Arabidopsis eFP Browser (Winter et al., 2007).

Ratio of *ACLA1/ACLB2* expression

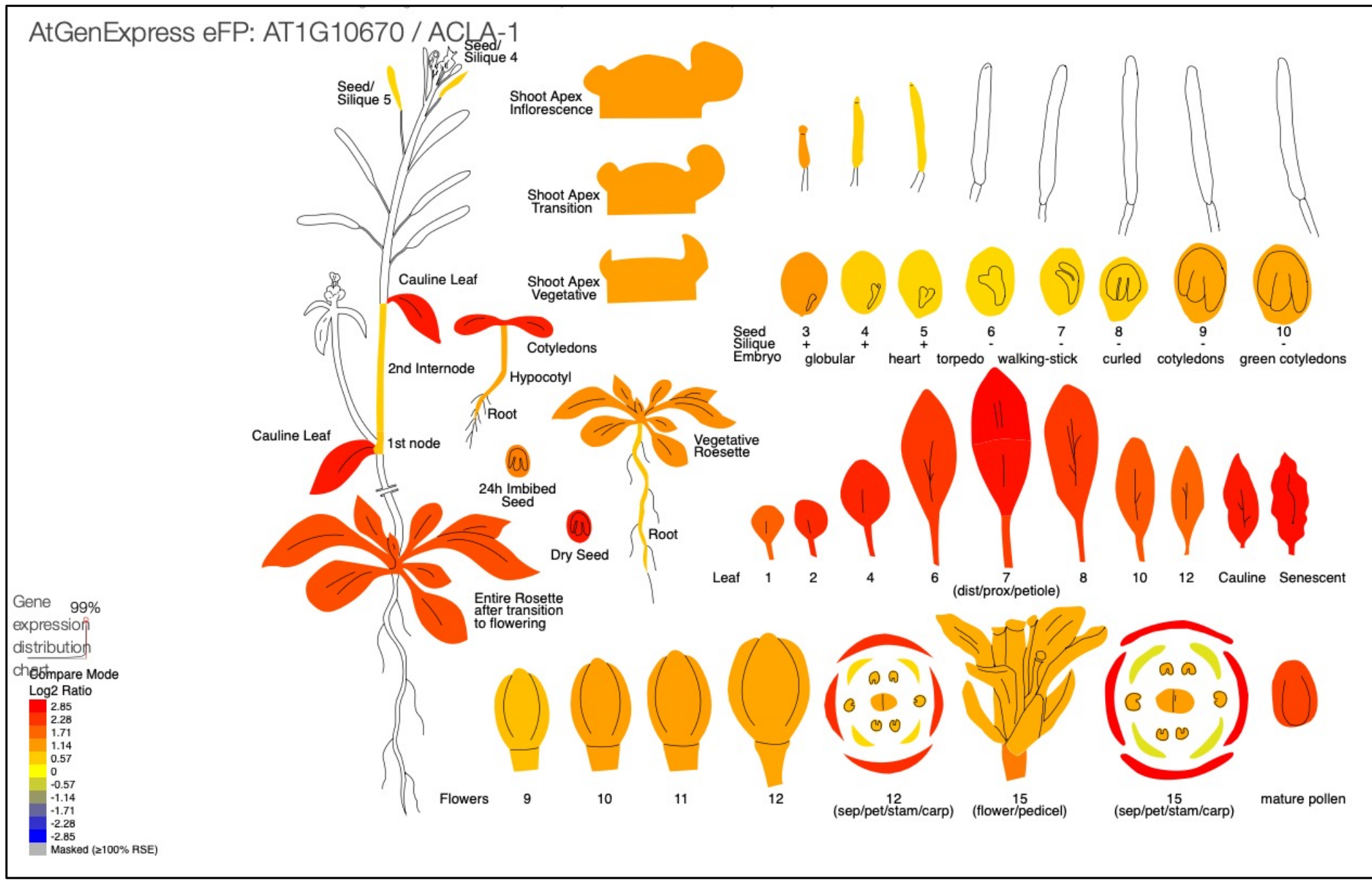

Ratio of *ACLB1/ACLB2* expression

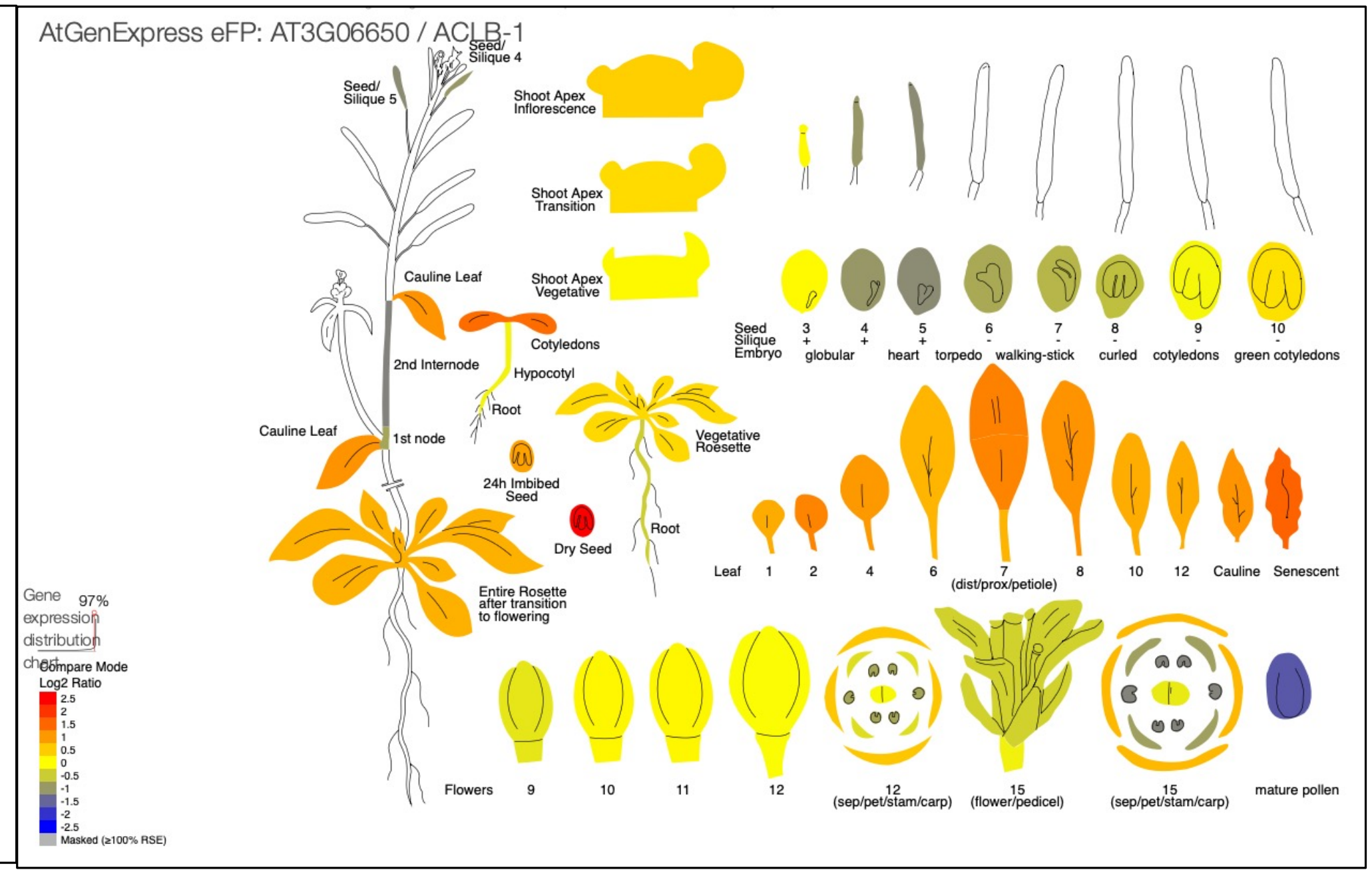

Supplemental Figure S7. Expression of *ACLA1* and *ACLB1* relative to the expression of *ACLB2*. Expression data were visualized at the Arabidopsis eFP Browser (Winter et al., 2007).

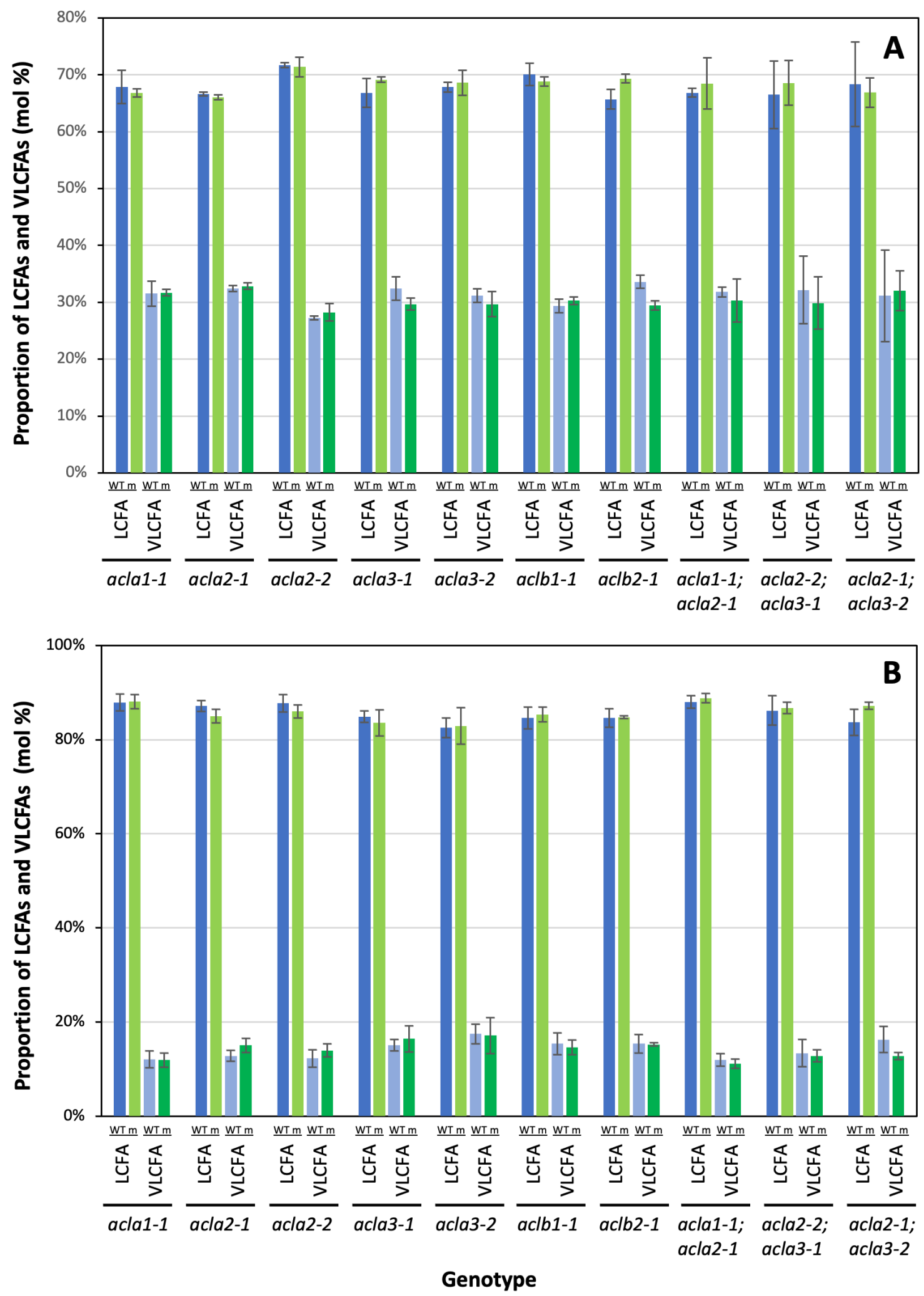

Supplemental Figure S8. Proportion of long chain fatty acids (LCFAs) and very long chain fatty acids (VLCFAs) in seeds (A) and roots (B). LCFAs are defined as fatty acids of up to 18-carbon chain length, and VLCFAs are fatty acids that are longer than 18-carbon chain length.
