# Supplementary material for: Functional redundancy and synthetic haploinsufficiency among Arabidopsis ATP-citrate lyase genes
Source: Front Plant Sci. 2026 Oct 9;17:1961717. doi: 10.3389/fpls.2026.1961717 (PMC13690957; doi:10.3389/fpls.2026.1961717)
Supplement: Supplementary file 2 [file Table5.docx]

**Supplemental Table S5**. Statistical analysis of allelism test between *acla3-1* and *acla3-2* alleles

| Genotypes of recovered progeny^1^ | | | | | | |  | Statistical test^2^ | | | | |
| --- | --- | --- | --- | --- | --- | --- | --- | --- | --- | --- | --- | --- |
|  |  |  |  |  |  |  |  | χ^2^ |  | α = 0.05 |  | d.f.^3^ |
| *acla3-2/acla3-2* |  | *acla3-2/acla3-1* | |  | *acla3-1/acla3-1* | |  |  |  |  |  |  |
| 6 |  | 31 |  | | | 10 |  | 5.47 |  | 5.99 |  | 2 |

^1^ Number of progeny of the indicated genotype recovered in the F2 generation from the cross *acla3-2/acla3-2* ♂ × *aca3-1/acla3-1* ♀, which tested allelism between the two *acla3* mutant alleles

^2^ Statistical χ^2^ test of the null hypothesis that the mutant allele segregates at the Mendelian ratio (1:2:1) for a recessive non-lethal mutation

^3^ d.f., degrees of freedom
